# Compilation of an economy-wide material flow database for 14 stock-building materials in 177 countries from 1900 to 2016^☆^

**DOI:** 10.1016/j.mex.2022.101654

**Published:** 2022-03-09

**Authors:** Barbara Plank, Jan Streeck, Doris Virág, Fridolin Krausmann, Helmut Haberl, Dominik Wiedenhofer

**Affiliations:** Institute of Social Ecology, BOKU Vienna; Schottenfeldgasse 29, Vienna 1070, Austria

**Keywords:** Material flow analysis, Resource use, Long-term analysis, Uncertainty assessment, Industrial ecology, Social metabolism

## Abstract

International datasets on economy-wide material flows currently fail to comprehensively cover the quantitatively most important materials and countries, to provide centennial coverage and to differentiate between processing stages. These data gaps hamper research and policy on resource use. Herein, we present and document the data processing and compilation procedures applied to develop a novel economy-wide database of primary stock-building material flows systematically covering 177 countries from 1900- 2016. The main methodological novelty is the consistent integration of material flow accounting and analysis principles and thereby addresses limitations in terms of transparency, data quality and uncertainty treatment. The database systematically discerns four processing stages from raw materials extraction, to processing of raw and semi-finished products, to manufacturing of stock-building materials. Included materials are concrete, asphalt, bricks, timber products, paper, iron & steel, aluminium, copper, lead, zinc, other metals, plastics, container and flat glass. The database is compiled using international and national data sources, using a transparent and consistent 10-step procedure, as well as a systematic uncertainty assessment. Apart from a detailed documentation of the data compilation, validations of the database using data from previous studies and additional uncertainty estimates are presented.

• Systematically compiled historical database of primary stock-building material flows for 177 countries.

• Consistent integration of economy-wide material flow accounting and detailed material flow analysis principles.

• Methodological enhancements in terms of transparency, data quality and uncertainty treatment.

## Graphical abstract

**Figure:**
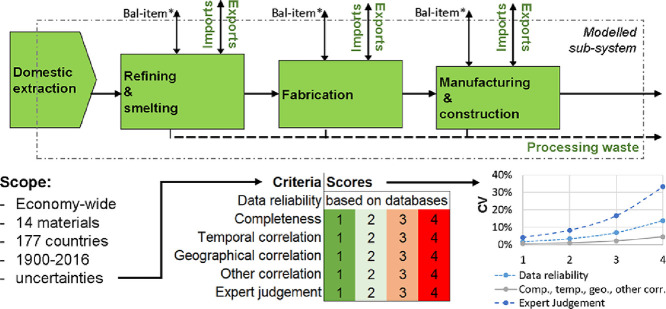

**Specifications table**

**Table:** 

Subject Area:	Environmental Science
More specific subject area:	Industrial Ecology
Method name:	Economy-wide material flow analysis for stock-building materials
Name and reference of original method:	• Fischer-Kowalski, M., Krausmann, F., Giljum, S., Lutter, S., Mayer, A., Bringezu, S., Moriguchi, Y., Schütz, H., Schandl, H., & Weisz, H. (2011). Methodology and Indicators of Economy-wide Material Flow Accounting. *Journal of Industrial Ecology, 15*(6), 855–876. 10.1111/j.1530–9290.2011.00366.x • Krausmann, F., Schandl, H., Eisenmenger, N., Giljum, S., & Jackson, T. (2017). Material Flow Accounting–Measuring Global Material Use for Sustainable Development. *Annual Review of Environment and Resources, 42*(1), 647–675. 10.1146/annurev-environ-102016–060726 • Wiedenhofer, D., Fishman, T., Lauk, C., Haas, W., & Krausmann, F. (2019). Integrating Material Stock Dynamics Into Economy-Wide Material Flow Accounting–Concepts, Modelling, and Global Application for 1900–2050. Ecological Economics, 156, 121–133. 10.1016/j.ecolecon.2018.09.010
Resource availability;	Supporting Information

## Method details

This article describes the methodological approaches used to compile the database presented in the article “From resource extraction to manufacturing and construction: flows of stock-building materials in 177 countries from 1900 to 2016”, which was recently published in the journal ‘Resources, Conservation and Recycling’ [142].

## 1 The model scheme–Quantified processes and flows

As theoretical and methodological background for the quantification of primary stock-building material flows, the herein presented database combines harmonized methods and principles from economy-wide material flow accounting [45,74] and material flow analysis [20,53].

**Fig. 1:**
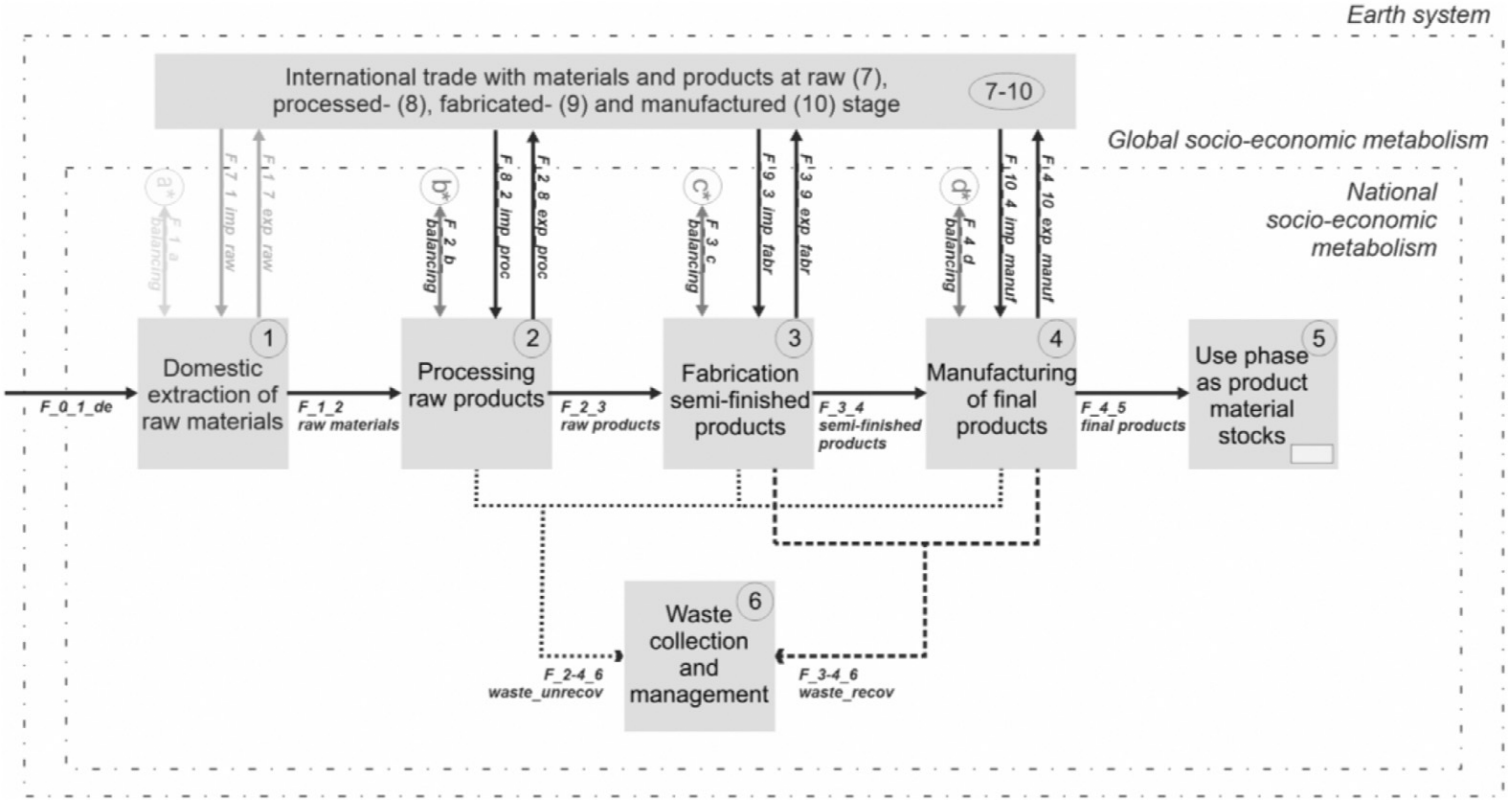
Process and flow scheme for the systematic definition of the herein presented data (definitions are given in Table 1). Boxes indicate processes, continuous lines indicate materials going to the next processing stage and dotted lines materials going to waste (recoverable and unrecoverable). Recovered material flows from latter processing stages are not reinstated in the system.

The systems definition shows the boundaries applied and processes discerned in this work (Fig. 1). Process 1 is defined as the *Domestic extraction of raw materials* and represents activities from mining and forestry sectors where natural resources are extracted directly from the environment (e.g. iron ore) and includes all used extraction, while excluding un-used extraction, as defined in economy-wide material flow accounting [45]. *Domestic extraction* follows a territorial principle, i.e., it accounts for all materials extracted within the national territory or each country, no matter where extracting companies are legally based. Process 2 is defined as the *Processing of raw products,* which constitutes the first step where raw materials are transformed and refined in usually highly standardized industrial processes to produce raw products (e.g. smelting ores into metals such as crude steel and separating them from gangue and extractive waste). Process 3 is defined as the *Fabrication of semi-finished products,* which are also typically fabricated in standardized ways, but are mostly clustered in other economic sectors and industries than raw processing (e.g. steel beams). The last process 4 is the *Manufacture of final products,* which usually involves more diverse and unstandardized industrial methods and yield a broad variety of final products (e.g. buildings, infrastructure, machinery, etc.). Process 5 (use phase) and 6 (waste management) are outside of the scope of this study and not further investigated.

Detailed definitions on the terms and calculations of all quantified parameters are given in Table 1. *Exogenous data* for process 2 (processing of raw products) was collected, as most comprehensive datasets were available at that stage (except for wood and paper, where production data on semi-finished products was collected), as well as trade data and factors for processing losses and wastes for each of these processing stages. All other model variables (*endogenous data*) were calculated by combining these exogenous variables as shown by the equations in Table 1. So far, imports and exports of raw materials (F_7_1, F_1_7) have not yet been quantified, but only derived global domestic extraction by applying global yield factors from previous studies, as explained in Section 9 [74]. When inconsistencies occurred due to ill-matched production and trade data, *balancing flows* for each processing step (a*, b*, c*, d*) were introduced, which represents the globally accumulated error of data matching. Details on data collection and processing can be found in Section 3.

**Table 1:** List of processes, flows and system variables.

Index	Definition	Compilation method
**Processes**		
**P1**	Extraction of raw materials	
**P2**	Processing of raw products	
**P3**	Fabrication of semi-finished products	
**P4**	Manufacturing of final products	
**P5**	Use phase of product material stocks	
**P6**	Waste collection, management and treatment	
**P7**	Trading of raw materials	
**P8**	Trading of raw products	
**P9**	Trading of semi-finished products	
**P10**	Trading of final products	
**Material input/output flows**		
**F_0_1**	Domestic extraction	See Section 9
**F_1_2**	Raw material output	Exogenous data
**F_2_3**	Output of raw products	F_1_2*(1-L2-W2) + F*_*8_2 – F_2_8
**F_3_4**	Output of semi-finished products	F_2_3*(1-L3-W3) + F*_*9_3 – F_3_9
**F_4_5**	Output of final products / primary gross additions to stock (GAS_prim_)	F_3_4*(1-L4-W4) + F*_*10_4 – F_4_10
**Material trade flows**		
**F_7_1**	Imports of raw materials	Not compiled
**F_1_7**	Exports of raw materials	Not compiled
**F_8_2**	Imports of raw products	Exogenous data
**F_2_8**	Exports of raw products	Exogenous data
**F_9_3**	Imports of semi-finished products	Exogenous data
**F_3_9**	Exports of semi-finished products	Exogenous data
**F_10_4**	Imports of final products	Exogenous data
**F_4_10**	Exports of final products	Exogenous data
**Processing wastes**		
**F_2–4_6**	Unrecoverable wastes from extraction, processing, fabrication and manufacturing	F_1_2*L2; F_2_3*L3; F_3_4*L4
**F_3–4_6**	Recoverable wastes from processing, fabrication and manufacturing	F_1_2*W2; F_2_3*W3; F_3_4*W4
**L2–4**	Unrecoverable waste shares of total production output	Exogenous data
**W3–4**	Recoverable waste shares of total production output	Exogenous data

Furthermore, the present database only quantifies primary material flows that stem directly from resources extracted from the environment in the same time period and do not include flows of secondary materials; the approach to exclude secondary materials from production and trade data is explained in Section 7. From primary inputs, furthermore losses and wastes occurring during the different processing stages were deducted, to identify only materials that directly stem from the environment for stock construction. *Unrecoverable processing wastes (waste_unrec)* represent the unrecoverable part of processing wastes that is treated further by waste management or dissipatedly lost to the environment. *Recoverable processing wastes (waste_rec)* represent the recoverable part of occurring wastes and are especially for metals usually designated as new scrap. As the goal of this work is to quantify primary material flows, recovered materials are not reinstated in the system but rather collected in waste collection and management.

The herein defined processing stages vary widely across the range of materials we consider in this work. We therefore defined production processes and product groups included at each processing stage in line with the material-specific scientific literature available. Definitions (and respective examples) at each of the processing stages for each of the 14 materials considered in this work are given in Table 2.

**Table 2:** Classification, definition and examples of materials and products at the four distinguished processing stages.

Material	Processing stages			
	(P1) Raw materials	(P2) Raw products	(P3) Semi-finished products	(P4) Final products
**Concrete**	Limestone, clay, sand and gravel	Cement, sand and gravel	Concrete	buildings, infrastructure
**Asphalt**	Crude oil, sand and gravel	Bitumen, sand and gravel	Asphalt	roads, infrastructure
**Bricks**	Clays and kaolin	Bricks	Articles of bricks	buildings, infrastructure
**Paper**	Industrial roundwood overbark	Industrial roundwood underbark	Primary paper and paperboard	Articles of paper and paperboard, printed matter
**Wood**	Industrial roundwood overbark	Industrial roundwood underbark	Sawnwood, wood-based panels, other industrial roundwood	Wood and cork manufactures
**Iron & steel**	Iron-based ore	Crude steel, casting iron	Semi-finished iron and steel products (plates, sheets, rails …)	Final iron and steel products (buildings, machinery, appliances …)
**Aluminium**	Bauxite	Primary aluminium	Semi-finished aluminium products (plates, tubes, pipes …)	Final aluminium products (Machinery, appliances, packaging …)
**Copper**	Copper ore	Primary copper	Semi-finished copper products (plates, tubes, cables …)	Final copper products (Machinery, electric appliances …)
**Lead**	Lead ore	Primary lead	Semi-finished lead products	Final lead products (batteries, accus …)
**Zinc**	Zinc ore	Primary zinc	Semi-finished zinc products and alloys (anti-corrosion agent …)	Final zinc products (machinery, infrastructure …)
**Other metals**	Metal ores	Metal content (in non-steel alloys)	Semi-finished other metal alloys	Final products from other metals
**Plastics**	Crude oil, natural gas	Thermoplastics, rubber, fibers	Semi-finished plastics and rubber products	Final products of plastics and rubber (plastics content)
**Container glass**	Limestone, silica sands, soda ash	Hollow/container glass	Containers and glassware	Final products of container glass (bottles, cups …)
**Flat glass**	Limestone, silica sands, soda ash	Flat glass	Flat glass	Final products of flat glass (windows, mirrors …)

## 2 Country selection, GDP and population data

In this dataset, 177 currently existing countries are discerned. Because country definitions changed over the last 120 years and data availability for some countries can be problematic, one consistent classification of countries for the entire time series was applied. Firstly, all countries which are currently not contended and have more than 300 000 inhabitants were selected, primarily because the excluded smaller countries have very poor data availability. Secondly, historically divergent country definitions were corrected, which stem from country fusions and dissolutions as well as other territorial changes to the best of our knowledge, based on a review of territorial changes throughout the 20th century. Historical country aggregates like the USSR were disaggregated into its successor states by back-casting shares of the earliest available datapoint of the historic aggregate to arrive at a contemporary country classification. The historic country aggregates that were corrected in most of the data sources were Belgium-Luxembourg, Czechoslovakia, Ethiopia incl. Eritrea, Serbia and Montenegro, Sudan, USSR and Yugoslavia. The final list of the 177 countries covered in this dataset can be found in Table 3.

**Table 3:** List of countries included in the novel database and several world regional groupings. Income levels are taken from the World Bank [139] classification for 2016 (limits in Gross National Income (GNI) per capita in US$)–*L*= low income (<=1005), LM= lower middle income (1006–3955), UM= upper middle income (3956–12,235), *H*= high income (>12,235). World regions from Wiedenhofer et al. [137] –IOW – Industrial Old World, INW – Industrial New World, FSU – Former Soviet Union, Asia, other, China, India, MENA – Middle East & Northern Africa, LACA – Latin America & the Caribbean, SSA – Sub-Saharan Africa.

Countries	UN Code ISO3166	Geographical region	MISO Region [137]	World-Bank Income level
**Afghanistan**	4	Southern Asia	Asia, other	L
**Albania**	8	Southern Europe	IOW	UM
**Algeria**	12	Northern Africa	MENA	UM
**Angola**	24	Sub-Saharan Africa	SSA	LM
**Argentina**	32	Latin America and the Caribbean	LACA	UM
**Armenia**	51	Western Asia	FSU	LM
**Australia**	36	Australia and New Zealand	INW	H
**Austria**	40	Western Europe	IOW	H
**Azerbaijan**	31	Western Asia	FSU	UM
**Bahamas**	44	Latin America and the Caribbean	LACA	H
**Bahrain**	48	Western Asia	MENA	H
**Bangladesh**	50	Southern Asia	Asia, other	LM
**Belarus**	112	Eastern Europe	FSU	UM
**Belgium**	56	Western Europe	IOW	H
**Belize**	84	Latin America and the Caribbean	LACA	UM
**Benin**	204	Sub-Saharan Africa	SSA	L
**Bhutan**	64	Southern Asia	Asia, other	LM
**Bolivia**	68	Latin America and the Caribbean	LACA	LM
**Bosnia and Herzegovina**	70	Southern Europe	IOW	UM
**Botswana**	72	Sub-Saharan Africa	SSA	UM
**Brazil**	76	Latin America and the Caribbean	LACA	UM
**Brunei**	96	South-eastern Asia	Asia, other	H
**Bulgaria**	100	Eastern Europe	IOW	UM
**Burkina Faso**	854	Sub-Saharan Africa	SSA	L
**Burundi**	108	Sub-Saharan Africa	SSA	L
**Cambodia**	116	South-eastern Asia	Asia, other	LM
**Cameroon**	120	Sub-Saharan Africa	SSA	LM
**Canada**	124	Northern America	INW	H
**Cape Verde**	132	Sub-Saharan Africa	SSA	LM
**Central African Republic**	140	Sub-Saharan Africa	SSA	L
**Chad**	148	Sub-Saharan Africa	SSA	L
**Chile**	152	Latin America and the Caribbean	LACA	H
**China**	156	Eastern Asia	China	UM
**Colombia**	170	Latin America and the Caribbean	LACA	UM
**Comoros**	174	Sub-Saharan Africa	SSA	L
**Congo**	178	Sub-Saharan Africa	SSA	LM
**Congo, DR**	180	Sub-Saharan Africa	SSA	L
**Costa Rica**	188	Latin America and the Caribbean	LACA	UM
**Côte d'Ivoire**	384	Sub-Saharan Africa	SSA	LM
**Croatia**	191	Southern Europe	IOW	UM
**Cuba**	192	Latin America and the Caribbean	LACA	UM
**Cyprus**	196	Western Asia	IOW	H
**Czech Republic**	203	Eastern Europe	FSU	H
**Denmark**	208	Northern Europe	IOW	H
**Djibouti**	262	Sub-Saharan Africa	MENA	LM
**Dominican Republic**	214	Latin America and the Caribbean	LACA	UM
**Ecuador**	218	Latin America and the Caribbean	LACA	UM
**Egypt**	818	Northern Africa	MENA	LM
**El Salvador**	222	Latin America and the Caribbean	LACA	LM
**Equatorial Guinea**	226	Sub-Saharan Africa	SSA	UM
**Eritrea**	232	Sub-Saharan Africa	SSA	L
**Estonia**	233	Northern Europe	FSU	H
**Ethiopia**	231	Sub-Saharan Africa	SSA	L
**Fiji**	242	Melanesia	Asia, other	UM
**Finland**	246	Northern Europe	IOW	H
**France**	250	Western Europe	IOW	H
**Gabon**	266	Sub-Saharan Africa	SSA	UM
**Georgia**	268	Western Asia	FSU	LM
**Germany**	276	Western Europe	IOW	H
**Ghana**	288	Sub-Saharan Africa	SSA	LM
**Greece**	300	Southern Europe	IOW	H
**Guadeloupe**	312	Latin America and the Caribbean	LACA	H
**Guatemala**	320	Latin America and the Caribbean	LACA	LM
**Guinea**	324	Sub-Saharan Africa	SSA	L
**Guinea Bissau**	624	Sub-Saharan Africa	SSA	L
**Guyana**	328	Latin America and the Caribbean	LACA	UM
**Haiti**	332	Latin America and the Caribbean	LACA	L
**Honduras**	340	Latin America and the Caribbean	LACA	LM
**Hong Kong SAR**	344	Eastern Asia	Asia, other	H
**Hungary**	348	Eastern Europe	IOW	H
**Iceland**	352	Northern Europe	IOW	H
**India**	356	Southern Asia	India	LM
**Indonesia**	360	South-eastern Asia	Asia, other	LM
**Iran**	364	Southern Asia	MENA	UM
**Iraq**	368	Western Asia	MENA	UM
**Ireland**	372	Northern Europe	IOW	H
**Israel**	376	Western Asia	MENA	H
**Italy**	380	Southern Europe	IOW	H
**Jamaica**	388	Latin America and the Caribbean	LACA	UM
**Japan**	392	Eastern Asia	IOW	H
**Jordan**	400	Western Asia	MENA	LM
**Kazakhstan**	398	Central Asia	FSU	UM
**Kenya**	404	Sub-Saharan Africa	SSA	LM
**Kuwait**	414	Western Asia	MENA	H
**Kyrgyzstan**	417	Central Asia	FSU	LM
**Laos**	418	South-eastern Asia	Asia, other	LM
**Latvia**	428	Northern Europe	FSU	H
**Lebanon**	422	Western Asia	MENA	UM
**Lesotho**	426	Sub-Saharan Africa	SSA	LM
**Liberia**	430	Sub-Saharan Africa	SSA	L
**Libya**	434	Northern Africa	MENA	UM
**Lithuania**	440	Northern Europe	FSU	H
**Luxembourg**	442	Western Europe	IOW	H
**Madagascar**	450	Sub-Saharan Africa	SSA	L
**Malawi**	454	Sub-Saharan Africa	SSA	L
**Malaysia**	458	South-eastern Asia	Asia, other	UM
**Maldives**	462	Southern Asia	Asia, other	UM
**Mali**	466	Sub-Saharan Africa	SSA	L
**Malta**	470	Southern Europe	IOW	H
**Martinique**	474	Latin America and the Caribbean	LACA	H
**Mauritania**	478	Sub-Saharan Africa	SSA	LM
**Mauritius**	480	Sub-Saharan Africa	SSA	UM
**Mexico**	484	Latin America and the Caribbean	IOW	UM
**Moldova**	498	Eastern Europe	FSU	LM
**Mongolia**	496	Eastern Asia	Asia, other	LM
**Montenegro**	499	Southern Europe	IOW	UM
**Morocco**	504	Northern Africa	MENA	LM
**Mozambique**	508	Sub-Saharan Africa	SSA	L
**Myanmar**	104	South-eastern Asia	Asia, other	LM
**Namibia**	516	Sub-Saharan Africa	SSA	UM
**Nepal**	524	Southern Asia	Asia, other	L
**Netherlands**	528	Western Europe	IOW	H
**New Zealand**	554	Australia and New Zealand	INW	H
**Nicaragua**	558	Latin America and the Caribbean	LACA	LM
**Niger**	562	Sub-Saharan Africa	SSA	L
**Nigeria**	566	Sub-Saharan Africa	SSA	LM
**North Korea**	408	Eastern Asia	Asia, other	L
**North Macedonia**	807	Southern Europe	FSU	UM
**Norway**	578	Northern Europe	IOW	H
**Oman**	512	Western Asia	MENA	H
**Pakistan**	586	Southern Asia	Asia, other	LM
**Panama**	591	Latin America and the Caribbean	LACA	UM
**Papua New Guinea**	598	Melanesia	Asia, other	LM
**Paraguay**	600	Latin America and the Caribbean	LACA	UM
**Peru**	604	Latin America and the Caribbean	LACA	UM
**Philippines**	608	South-eastern Asia	Asia, other	LM
**Poland**	616	Eastern Europe	IOW	H
**Portugal**	620	Southern Europe	IOW	H
**Puerto Rico**	630	Latin America and the Caribbean	LACA	H
**Qatar**	634	Western Asia	MENA	H
**Réunion**	638	Sub-Saharan Africa	SSA	LM
**Romania**	642	Eastern Europe	IOW	UM
**Russia**	643	Eastern Europe	FSU	UM
**Rwanda**	646	Sub-Saharan Africa	SSA	L
**Saudi Arabia**	682	Western Asia	MENA	H
**Senegal**	686	Sub-Saharan Africa	SSA	L
**Serbia (incl. Kosovo)**	688	Southern Europe	IOW	UM
**Sierra Leone**	694	Sub-Saharan Africa	SSA	L
**Singapore**	702	South-eastern Asia	Asia, other	H
**Slovakia**	703	Eastern Europe	FSU	H
**Slovenia**	705	Southern Europe	FSU	H
**Solomon Islands**	90	Melanesia	Asia, other	LM
**Somalia**	706	Sub-Saharan Africa	SSA	L
**South Africa**	710	Sub-Saharan Africa	SSA	UM
**South Korea**	410	Eastern Asia	IOW	H
**South Sudan**	728	Sub-Saharan Africa	SSA	L
**Spain**	724	Southern Europe	IOW	H
**Sri Lanka**	144	Southern Asia	Asia, other	LM
**Sudan**	729	Northern Africa	SSA	LM
**Suriname**	740	Latin America and the Caribbean	LACA	UM
**Swaziland**	748	Sub-Saharan Africa	SSA	LM
**Sweden**	752	Northern Europe	IOW	H
**Switzerland**	756	Western Europe	IOW	H
**Syria**	760	Western Asia	MENA	LM
**Taiwan**	158	Eastern Asia	Asia, other	H
**Tajikistan**	762	Central Asia	FSU	LM
**Tanzania**	834	Sub-Saharan Africa	SSA	L
**Thailand**	764	South-eastern Asia	Asia, other	UM
**The Gambia**	270	Sub-Saharan Africa	SSA	L
**Timor-Leste**	626	South-eastern Asia	Asia, other	LM
**Togo**	768	Sub-Saharan Africa	SSA	L
**Trinidad and Tobago**	780	Latin America and the Caribbean	LACA	H
**Tunisia**	788	Northern Africa	MENA	LM
**Turkey**	792	Western Asia	IOW	UM
**Turkmenistan**	795	Central Asia	FSU	UM
**Uganda**	800	Sub-Saharan Africa	SSA	L
**Ukraine**	804	Eastern Europe	FSU	LM
**United Arab Emirates**	784	Western Asia	MENA	H
**United Kingdom of Great Britain and Northern Ireland**	826	Northern Europe	IOW	H
**United States of America**	840	Northern America	INW	H
**Uruguay**	858	Latin America and the Caribbean	LACA	H
**Uzbekistan**	860	Central Asia	FSU	LM
**Venezuela**	862	Latin America and the Caribbean	LACA	UM
**Vietnam**	704	South-eastern Asia	Asia, other	LM
**Yemen**	887	Western Asia	MENA	LM
**Zambia**	894	Sub-Saharan Africa	SSA	LM
**Zimbabwe**	716	Sub-Saharan Africa	SSA	L

A comprehensive population dataset for calculating per-capita values and back-casting specific material flows was derived by combining several sources. For 1950–2018, data from the UN population division [113] was used, which is comprehensive for all countries in the dataset. For early years, 1950 UN population numbers were extrapolated with country-level trends available from the historical population datasets provided by the Maddison project [92] and the CLIO Infra project [44]. The Maddison time series are continuous for many countries back until at least 1900, while the CLIO Infra data source only provides data every ten years. Therefore, Maddison trends were used whenever possible, supplemented with data points from the CLIO Infra dataset. All residual data points were linearly interpolated. For historic country aggregates, population accounts were disaggregated along the same procedure as applied for material flow data, which is explained in Section 3/Step 3. If neither Maddison nor CLIO Infra data was available, which was the case for 10 small countries in the database, the respective world-regional trend to back-cast 1950 UN population numbers was used.

For gross domestic product (GDP), the Maddison database [92] is the most comprehensive data source providing GDP per capita in international Dollars of 1990 (i.e. in Purchasing Power Parity) for almost all countries back until 1950 and an extensive selection of countries for earlier years. GDP accounts for the rest of the countries were not estimated as they are less stable than population accounts, and therefore GDP was only calculated by multiplying per-capita values with population for a selection of countries. After 1950, no GDP data was available for 24 countries of the dataset; before 1950, no data was available for 82 countries. Therefore, investigations with GDP were only conducted after 1950 to ensure sufficient coverage.

## 3 Data collection, processing and estimation procedures

To develop a consistent material flow dataset for 14 materials covering the entire time period of 1900 to 2016, systematic and standardized procedures were developed along the following ten steps.

**Step 1 – Identify, assess and collect relevant data sources:** All relevant data available from different data sources was gathered for each material category and datasets were analyzed according to which indicators come closest to production of raw products or semi-manufactured products, or in case calculated production flows from other available indicators. In addition, relevant trade flows were identified and categorized, different data sources compared and the one with the highest country coverage chosen. Details on the data sources utilized are described in Section 4.

**Step 2 - Harmonize datasets to the common classification within our database structure:** The following data processing steps were applied to the reported data to ensure harmonization with our classifications and avoid loss of existing information:

• Simple unit conversions (e.g. pound to kg),
• *Re*-estimations based on factors (e.g. volume to weight using standard factors),
• Sequencing of data from datasets with overlapping time periods by always using the latest available dataset,
• Harmonization of country names,
• Combination of different data sources to one comprehensive dataset.

**Step 3 - Correct major changes of country definitions over the studied time period:** To arrive at a consistent set of 177 contemporary nation states, major changes in country territory and definitions happening throughout the 20th and 21st century were corrected by applying the following two standard procedures:

○ Country fusion: aggregate previous countries or territories into the newly formed country
○ Country dissolution: disaggregate material flow data reported for the historic country aggregate by shares of the earliest available datapoints of the sum of the historic aggregate for each successor state (used for e.g. the USSR, former Yugoslavia, Czechoslovakia, Sudan …)

**Step 4 – Interpolating data gaps & plausibility checks**: After collecting and cleaning datasets, various gaps, outliers and data errors remain to be corrected. A conservative but systematic data cleaning approach was developed along three rationales, which are also reflected in the uncertainty scoring of the respective datapoints.

• Outlier removal: Single outliers in country time series were replaced if both neighbours were larger or smaller than 2.5 times the single value for values larger than 10 kt and 10 times for lower weight values, or the absolute value was larger than trade from 1962 to 2018 divided by 3. The margins for outlier removal were chosen after an initial assessment on which margins remove very large outliers but otherwise leave original data largely as reported. In addition, several obvious outliers in production and trade data have been removed based on expert appraisal. Outliers lying in-between two datapoints were replaced by linear interpolation, outliers at the end of a time series were replaced using the appropriate method for extrapolation or back-casting explained in Step 5.
• Interpolation: Frequently there are smaller gaps of a few years within otherwise reported data. These gaps were interpolated linearly. For interpolated data points, uncertainty was scored based on the distance to the nearest available datapoint (for details on uncertainty scoring see chapter 10).
• Plausibility checks: Further literature screenings on plausibility of long-term time series trends was conducted. Background information was compiled that informed manual data cleaning as wells as estimation methods.

**Step 5 – Back-casting and extrapolating data for non-available years:** Data availability across the 14 materials differs: some datasets date back to early 20th century, while others start as late as 1970 and sometimes time series do not continue until 2016 (see Section 6 for details). The following simple principles were utilized for back-casting and extrapolating data for those countries where the data does not cover the entire time period of 1900 to 2016.

• Extrapolation: For specific countries and materials (especially cement and bitumen), reported data ends either 2014 or 2015. Data was then extrapolated with the average annualized growth rates of the last available 4 years. This approach has only been applied after 2010 to avoid the economic crisis bias. In certain cases of time series ending earlier, data was extrapolated with GDP trends from the Maddison Project Database [92].
• Back-casting: Stock-building material use was modelled into the past using additional data sources or expert judgements, to have information for very early years. For each material, cut-off values were defined, which were considered too small to conduct back-casting procedures (described further in Section 6 for each material separately). Trade data was back-casted for all processing steps back until 1900 with a standardized approach using growth rates of global trade flows (constant US$) sourced from Federico and Tena-Junguito [43]. Production data was back-casted using one or a combination of the following approaches:
  ○ If additional data was available on the extraction of the raw materials of a product (e.g. crude oil production for bitumen), time series were back-casted with these trends for certain available years and countries. Further information when and where this approach was applied can be found below for each material separately.
  ○ Decide on a reasonable socio-technical “starting point” for the use of the material, then back-cast using GDP trends (if available) or linearly interpolate to the latest available datapoint. This applies to concrete, asphalt, plastics, aluminium, chromium, manganese.
  ○ If the use of the material is driven by industrialization (e.g. steel, copper, lead, zinc, glass) [74], data was back-casted with GDP trends from the Maddison Project Database [92].
  ○ If the material is also used for subsistence (e.g. wood, paper, bricks) [74], data was back-casted with population trends provided by the UN [113], the Maddison and the CLIO Infra project [44,92].

**Step 6 – Estimations applied for materials and countries with very fragmented data:** Especially for asphalt, wood, bricks, container and flat glass, data quality was still insufficient after the previous steps. Additional materials-specific estimation procedures were applied for each material, to arrive at full temporal and country coverage. These are explained in chapter 6.

**Step 7 – Developing uncertainty estimates for each datapoint:** Scoring of the reliability of data sources and estimation methods based on an evaluation framework proposed by Laner et al. [75] translating to normally distributed standard deviations for each datapoint. In addition, it was surveyed how many of the datapoints have been estimated as outlined above in comparison to the datapoints coming from data sources and how much these estimates account for of the total mass. Further information on the implementation of the uncertainty assessment and detailed results for each material can be found below in chapter 10.

**Step 8 – Deriving production outputs by subtracting processing waste:** To consider the amounts that are unrecoverable or recoverable waste arising during processing, production estimates on each processing stage were multiplied with recoverable and unrecoverable waste rates to derive production outputs. Waste rates were compiled in a literature review and are given below in chapter 8.

**Step 9 – Deriving apparent consumption estimates:** For each processing stage, apparent consumption estimates were calculated by adding net-trade balances (imports minus exports) to production outputs. Uncertainty estimates of consumption estimates follow Gaussian error propagation rules.

**Step 10 – Creating a balancing item to deal with inconsistencies in the database:** Apparent consumption estimates can turn out negative due to missing production/import data or overestimation of exports. If consumption turned out negative, these values were linearly interpolated and differences between the new (interpolated) and the old (negative) values in a global balancing item summed up. In addition, these estimates were considered in our uncertainty assessment as further interpolations (for uncertainty scoring see Section 10) and their uncertainties were added to the uncertainties derived from Step 7. In addition, the global net-trade balance mostly does not sum up to zero due to several reasons: not all countries are covered in the database, classifications of products can vary between countries, several data adjustments were applied. As it is not possible to solve this problem on a bilateral trade level, the mismatch of trade data in the global balancing item of the consumption accounts was also accounted for, which can be understood as the “accumulated error” of production and trade data. The total global accumulated error from all mis-matching mass-balances is shown in Section 10.6.

Balanced consumption estimates (as derived from step 10) of each processing stage are then the inputs for the next processing stage and Steps 8–10 will be repeated until finally the primary gross additions to stock (GAS_prim_) for each material could be calculated.

## 4 Data sources

Some of the materials are consistently covered by international databases (e.g. cement, paper, iron and steel) while other can only be found in data sources showing severe reporting gaps for countries and time periods (e.g. bricks, bitumen, glass). For each material separately, relevant international databases and country-level scientific literature was reviewed to gather as many reported datapoints as possible. Which major databases to use was decided based on their country and time coverage and the definition of the reported production flows which comes closest to production of raw products or semi-manufactured products.

If the databases also reported trade flows, these were compiled as well. Most of the trade flows were collected from the UN Comtrade database and the reported products categories were further divided into processing stages (see Sections 1 & 5). In addition to international databases, we compiled data from scientific literature to complement and validate international databases Table 4. gives an overview on which processing stages have been covered by which data sources, where flows were assumed to be zero (no data available), and where flows could either be derived from mass-balancing previous flows or from estimation procedures.

**Table 4:** Data compilation methods and main data sources distinguished along processing stages. References for all data sources will be listed in Section 6 below. mass-bal.= mass-balancing of previous flows; zero= no data available, assumed zero; estimate= estimation/gap-filling.

*Material*	Input flow data to processing stages					
	(2) Raw products		(3) Semi-finished products		(4) Final products	
	*Production*	*Trade*	*Production*	*Trade*	*Production*	*Trade*
**Cement**	Cembureau	Cembureau	mass-bal.	Comtrade	mass-bal.	zero
**Bitumen**	IEA, UNICPS	IEA	mass-bal.	zero	mass-bal.	zero
**Bricks**	UNICPS	Comtrade	mass-bal.	zero	mass-bal.	zero
**Paper**	FAO	FAO	FAO	zero	mass-bal.	Comtrade
**Wood**	FAO	FAO	FAO	zero	mass-bal.	Comtrade
**iron & steel**	WSA, [97]	Comtrade	mass-bal.	Comtrade	mass-bal.	Comtrade
**aluminium**	WBMS, BGS	Comtrade, BGS	mass-bal.	Comtrade	mass-bal.	Comtrade
**Copper**	WBMS, BGS	Comtrade, BGS	mass-bal.	Comtrade	mass-bal.	Comtrade
**Lead**	BGS	Comtrade, BGS	mass-bal.	Comtrade	mass-bal.	Comtrade
**Zinc**	BGS	Comtrade, BGS	mass-bal.	Comtrade	mass-bal.	Comtrade
**Other metals**	BGS	BGS	mass-bal.	zero	mass-bal.	zero
**Plastics**	IEA	Comtrade	UNICPS	Comtrade	mass-bal.	Comtrade
**Container glass**	UNICPS, glassgobal	Comtrade	mass-bal.	Comtrade	mass-bal.	zero
**Flat glass**	UNICPS, glassgobal	Comtrade	mass-bal.	Comtrade	mass-bal.	zero
**Sand & gravel**	estimate	zero	mass-bal.	zero	mass-bal.	zero

## 5 Trade data compilation

Countries increasingly depend on trade to obtain resources, materials and products [48,108]. Therefore, the quantification of material flows which are consumed within a country requires a detailed account of materials in traded commodities. Physical trade flows for all materials were therefore assessed and compiled along the four processing stages as described in Section 2 (raw materials, raw products, semi-finished and final products). The database draws on trade information for 347 commodities, distinguishing content for 10 materials, for all countries worldwide for the period 1962–2018. Commodities were divided into the four processing stages of the system definition (see Section 1) according to their labels (see Table 5).

**Table 5:** List of SITC1 and SITC3 commodities classified into the four processing stages explained in section 1.

Processing stage	SITC1 codes (values in brackets SITC3 codes)
Raw products	242,244,26621,26622,26632,5811,5812,58131,58191,58199,664,671,672,679,6821,6841,6851,6861
Semi-finished products	2312,243,26623,26633,5332,53332,59959,59974,59975,59991,59994,59999,6112,6123,62101,62102,62103,62104,62105,631,641,65161,5162,65163,65164,65165,65191,65194,65229,65351,65352,65361,65362,6537,65391,65395,65401,65402,65403,65404,65405,65542,65543,65545,65546,65582,65583,65591,65592,65741,65742,66182,66183,665,673,674,675,676,677,678,68221,68222,68223,68224,68225,68226,68421,68422,68425,68426,68521,68522,68524,68621,68622,68623,69311,69312,69313,6932,69331,69332,69333,69341,69342,69343,2313,26631,65171,65172,65174
Final products	53333,53334,53335,54191,5530,57111,57112,5712,5713,5714,6121,6122,6291,6293,6294,62998,62999,632,633,642,65406,6551,65541,65561,65562,65563,65571,65572,65581,6561,6562,6566,65691,65692,6575,6576,6623,6624,66362,66391,6911,6912,6913,69211,69212,69213,69221,69222,69231,69232,69411,69412,69421,69422,695,696,69711,69712,69721,69722,69723,6979,6981,6982,6983,6984,6985,69861,69862,6988,69891,69892,69894,69896,69897,7111,7112,7113,7114,7115,7116,7117,7118,7121,7122,7123,7125,7129,7141,7142,7143,7149,715,717,718,7191,7192,7193,7194,7195,7196,7197,7198,7199,722,723,724,72501,72502,72503,72504,72505,726,7291,7292,7293,7294,7295,7296,7297,7299,7311,7312,7313,7314,7315,7316,7317,7321,7322,7323,7324,7325,7326,7327,732873291,73292,73311,73312,7333,7334,7341,73491,73492,7351,7353,7358,7359,8121,8123,81241,81242,81243,8210,8310,84111,84112,84113,84114,84121,84122,84123,84125,84126,84129,84141,84142,84143,84144,84151,84152,84153,84154,84159,8416,84202,85101,85102,85103,85104,861,86241,86242,8911,8912,8914,8918,8919,892,8930,894,8945,8951,89521,89522,89523,89592,89593,89594,89711,89713,89714,8972,89924,89927,89933,89934,89935,89941,89942,89943,89951,89952,89953,89954,89956,89957,89961,89962,89993,89997,89998,89999,95101,95102,95103,95104,95105,95106
Raw materials and scrap	2511, 2820, 28402, 28404, 28406, 28407, (579, 66411)

For process 2, curated materials-specific data sources were used such as Cembureau (cement [30]), IEA (bitumen [61]), FAO (solidwood and paper [42]) and BGS (metals [17]) (summary in Table 4). Because trade data from these databases in most cases only reports material flows for raw products and/or do not distinguish raw and semi-finished products properly (e.g. crude steel and steel pipes), we utilized data from the United Nations Commodity Trade Statistics Database (UN Comtrade) [114] to quantify physical trade for processes 3 and 4. In order to include the trade of materials contained in final products and to fill the gaps of the above mentioned data sources for other processing stages, trade in commodities was additionally obtained from the UN Comtrade database.

Trade in physical units is only reported in ca. 90% of data entries, compared to monetary trade values. To improve the country and time coverage of physical trade data from UN Comtrade, the data was corrected following procedures from [35,98], where we:

1. Downloaded bilateral commodity trade data for the commodities listed in Table 5 in the SITC1 classification for all countries from the UN Comtrade application programming interface (API).
2. Computed the average of double reported physical and monetary commodity trade flows (for flows reported twice, once as export of country A and once as import of country B).
3. Calculated a global weighted average price for each commodity and year with available monetary and mass trade figures (weighted by mass of commodity trade).
4. Manually checked the time-series of global average prices per commodity for consistency: for some years and commodities few trade entries were available which led to large deviations of average prices from other values in the time series. To increase consistency, values that deviated more than 10 times from the average of the two directly neighbouring years were substituted by the neighbours’ average.
5. Used the global average price per commodity and year to fill the gaps of physical trade where monetary, but no physical commodity trade figures were given (divided monetary trade by average price). In step five a total of 4 129 628 out of 52 339 158 datapoints was added (8.6%).
6. Removed outliers in bilateral trade data: Trade flows per commodity were grouped per exporter-importer-pair and sorted from 1962 to 2018. Afterwards, local extrema were identified (comparing trade mass for year t with the years *t* + 1 & t-1). In the case that the deviation of the extrema was larger than the boundaries given in Step 4 in Section S3, the extrema was substituted with the average of its neighbours. This procedure was repeated by comparing the average of two years to the direct neighbours of these datapoints. If the deviation was larger than the proposed boundaries, both values were substituted by interpolation. The datapoints added in Step 5, together with removing outliers in Step 6, contributed to covering an additional 2% of material mass in globally traded commodities for the whole period 1962–2018.
7. Computed commodity imports and exports per country and the associated material imports and exports by multiplication with material intensity information from ([80,93,96,98]; [129].). Material intensities were applied as time-constant values, except for the materials iron & steel and aluminium, for which intensities of commodity SITC 7321 (‘Passenger motor cars, other than buses’) were considered dynamic according to information from Liu & Müller [79]; and plastics, for which material intensities in traded commodities not reflecting raw plastic materials (e.g. plastic resins) were assumed to decrease from 2000 back to 1962 according to the trend in global plastic polymer resin and fibre production from Geyer et al. [47]. Except for the commodity codes for which material content information was reported in the prior studies, only commodities for which a material content of close to 100% of the respective material could be assumed were considered (e.g. 100% wood for “wood manufactures, nes”). Material contents used are given in the Supporting data file attached to this article. After calculating material content, ca*.* 87% of the original commodity trade is reflected in the dataset on material trade.
8. Removed outliers in imports and exports for country time-series: as still some larger outliers made it through previous steps, again values were compared to their two direct neighbours in the time series. If both neighbours were larger than, 10 times the value in question for flows lower than 1 Megaton per year (Mt/yr), or 2.5 times for values larger than 1 Mt/yr these values were substituted by the average of both neighbours. These boundaries appeared to be appropriate for removing large outliers while still preserving year-to-year variations in trade that seemed sensible to us.
9. The SITC1 classification was chosen in order to span the years back to 1962. However, not all flows of interest were available via this classification type. Therefore, in addition trade in plastic and glass waste and scrap were downloaded in classification SITC3 without any further manipulation (SITC3 codes 579 & 66,411).
10. After all these modifications, UN Comtrade data has been further processed as described above in Section 3, including further interpolations and manual outlier removals, for each compiled dataset on imports and exports of raw, semi-finished, final and waste products of 10 materials.
11. Furthermore, for trade before 1962 (which is the earliest year for which data from UN Comtrade was available), all trade datasets were back-casted using growth rates of global trade flows (constant US$) sourced from Federico and Tena-Junguito [43] until 1900, before we assumed trade flows to be negligible (see Section 3).
12. As a last step, uncertainties were assessed for all further data preparation steps as described in Section 10. Uncertainties of the modifications described in points 1–8 in this section could not be considered.

Following the principle of the conservation of mass, the global sum of imports and exports should be zero. The combination of trade data sources in our study could not always hold up to that and for some materials, the global net-trade balance is unequal to zero due to several reasons: trade customs declarations are sparsely registered in some countries, re-exports and re-imports are often not taken into account, imports for sales on unofficial markets or for military and defence purposes are not recorded in official statistics, our database does not cover all countries worldwide (but only the 177 presented in Section 2), products may be classified differently across countries and some data was estimated by several sources, including own estimations. Solving this problem on a bilateral trade level is not possible due to missing data and indications. Therefore, the net-trade balance was counterbalanced in the global balancing item of our consumption accounts (see Section 3).

## 6 Detailed documentation for each material

For each material, all data has been processed as described in Sections 3 and 5. Details on diverging data-processing and estimation procedures between the different materials are described in the following section. Details on uncertainty scoring of different data sources and estimation procedures presented in the following will be provided in the specially established Section 10 below.

### 6.1 Concrete

Concrete is the most widely used construction material and is composed of sand and gravel bonded together by fluid cement that hardens over time. Data on cement production and trade was mainly sourced from World Statistical Reviews of the European Cement Association (CEMBUREAU) for the years 1913–1995, 1996–2003, 1999–2009, 2001–2010 and 2004–2014 [26], [27], [28], [29], [30]. Data coverage in the provided datasets is very high, with data points for 165 out of the 177 countries investigated herein. Therefore, we did not additionally estimate cement production for missing countries. Statistics from CEMBUREAU refer to both cement and clinker, as countries usually do not differentiate between the two. For the two most recent years 2015 and 2016, we additionally added data from the latest CEMBUREAU Activity Report [31] for the main world cement producers and extrapolated data for all other countries using average growth rates of 2010–2014.

We classified cement production and trade flows reported by CEMBUREAU as raw products (p2 as defined in Table 2) and additionally added UN Comtrade data for trade in concrete components (p3). As we define final concrete products to be buildings and infrastructures, we assume no trade in final products. We define concrete as semi-finished product (p3) made out of the raw products cement and sand and gravel. We estimated the amount of sand and gravel necessary for concrete production by applying a ratio of cement-to-sand and gravel of 1:5, which is commonly used throughout the literature (e.g [24,74]).

Following previously established procedures for back-casting material flows using technological “starting points” and economic growth as proxies [74,135], we back-casted cement production using GDP growth rates for the years previous to 1913 if the latest reported datapoint is higher than 50 kt per year. The use of concrete in construction and therefore the use of modern Portland cement took off at around 1850 [34], which we therefore set as technological starting point and linearly reduced cement production from 1900 values to zero in 1850, assuming that all previously occurring historical uses of cement are negligible amounts for the purposes of this study.

### 6.2 Asphalt

Asphalt is produced from sand and gravel combined with bitumen, a refined petroleum product, and is primarily used for surfacing of roads and for roofing material. Data on bitumen production and trade was primarily sourced from the World Energy Balance database of the International Energy Agency [61] and from UNICPS [116,117]. The IEA database contains detailed harmonized information on energy and fossil fuel production and consumption for 180 countries and regions worldwide back until 1971 and for EU countries back until 1960. The 2017 dataset only contains data from 1960 to 2015, so we had to estimate 2016 values using average growth rates of 2011–2015. In addition to IEA data, we also used data on bitumen production from the United Nations Industrial Commodity Production Statistics database (UNICPS) [116,117], which does not have the same level of data quality as IEA data (confidentiality restrictions, differing product definitions, insufficient data collection) but reports data until 1950. When no IEA data was available (mostly between 1950 and 1970), we therefore used UNICPS data.

Data quality and coverage for country-level bitumen production and trade is intermediate and several corrections and estimations were necessary. IEA reported bitumen production flows for 91 countries in the country sample. Imports are insufficiently covered as they are only reported for 98 countries. Asphalt is used for road paving almost everywhere nowadays and over the last decades. We therefore additionally estimated bitumen production for 46 major countries based on per-capita values of countries in the same GDP decile in 2015, i.e., a classification of countries based on their per-capita GDP values in 2015.

Back-casting for bitumen production for the years prior to 1950 was done as follows: For 20 major countries, trends of historic oil production until 1800 can be found in Podobnik [101], which we used to back-cast bitumen production until 1900. When no such historical oil production data was available and the last reported datapoint was higher than 3 kt/year, we back-casted bitumen production using GDP growth rates until 1900. For some early industrializing countries like the USA, UK, France, Germany, USSR and Japan, Kern & Mayländer [68] report that asphalt road pavements started to be widely used around 1870. We therefore assumed a technological starting point in 1870 and linearly reduced bitumen production flows from 1900 to zero in 1870 or – if historical oil production data was available – linearly reduced the bitumen share of oil production from 5% in 1900 to 0% in 1870.

We classified bitumen production and trade flows as raw products (p2) and assume no trade in asphalt concrete products (p3) or final products like roads or roofing material (p4). Asphalt, which is made out of bitumen and sand and gravel, was classified as semi-finished product (p3). We estimated the amount of sand and gravel used for asphalt production by applying a ratio of bitumen-to-sand and gravel of 1:19, which is used throughout the literature (e.g [74,89]).

### 6.3 Bricks

Bricks produced from clay are used to build walls, pavements and other elements of masonry. Data on construction bricks made of clay was primarily sourced from the United Nations Industrial Commodity Production Statistics database (UNICPS) [116,117], (codes 37,350–1 A and 37,350–1 B).

Clay bricks are produced in different types and classes. To convert reported numbers from different statistics into mass of bricks, conversion factors were used. We used as volume conversion factor 0.0017m^3^ per unit of brick [71] and for conversion into mass an average factor of 2.84 kg per unit of brick derived from different sources (see Table 6).

**Table 6:** Mass conversion factors and their sources used to derive the average applied to bricks.

Source	unit brick [kg]
[126] (China)	3.20
[112] (UK)	2.77
[71]	2.50
[73]	4.20
[25]	2.75
[25]	2.50
[91] (South Africa)	3.60
[111] (USA)	1.80
[37] (Nepal)	2.00
[37] (Bangladesh)	3.50
[110] (India)	2.22
[37] (India)	3.00
**Average factor used**	**2.84**

Because data quality and coverage are generally very low for bricks, only data rows for countries reporting >10 datapoints were included. In addition to that, data from scientific literature and country-specific reports were used to compile time series for the UK [112], the USA [111], Japan [87], Austria [70], China [126] and India [37,42,110].

As the global dataset was not complete after inclusion of these data sources, linear interpolations were made wherever data rows showed gaps and some outliers had to be corrected. Extrapolations from values reported for past years up to 2016 were done by using the last reported value of t/cap and multiplying it with the country's population. For countries that did not report any data, estimates were derived by calculating an average of t bricks/capita from all countries that reported data grouped into high-income and low-income countries by using the classification of World Bank valid for 2016 [139]. The according value (low/high-income average) in t/cap was assigned to all countries that did not report any data and multiplied by population for every year.

Back-casting for bricks was done depending on population development, because bricks are a traditional building material assumed to be used rather independently of industrialization developments or fossil fuel use [71]. Therefore, in order to estimate bricks for early years, the first reported data point was converted into a value of t/capita and constantly back-casted until 1820 using population trends.

We classified bricks production and trade flows as raw products (p2) and assume no trade in articles of bricks (e.g. walls or ovens) (p3) or final products like buildings (p4).

### 6.4 Wood & paper

In this dataset, we only consider wood products and paper which accumulates as socio-economic material stocks, e.g. wood processed to sawnwood, wood-based panels and wood products as well as paper and exclude all wood use for fuel. Data has primarily been sourced from the FAO statistical database on food and agriculture [42], which comprehensively covers all major forestry production and trade flows for over 245 countries and territories worldwide back until 1961. Data coverage seems to be complete for paper and paperboard production and trade, no data can be found only for two countries in the sample and consumption estimates are consistent.

Data on wood products has been combined from reported data on sawnwood, wood-based panels and other industrial roundwood, which are usually reported in volume measures and were converted to weight using IPCC factors [63]. The category “other industrial roundwood” is typically used for the production of poles, piling, posts, fencing, pit props, tanning, etc. and is therefore included [63]. Data coverage on wood production is rather complete, covering 153 countries of the sample. To increase completeness even further, we estimated wood production for another six countries by using average shares of sawnwood and wood-based panels production of industrial roundwood production reported by FAO. We identify the residual 19 countries without solid-wood production to be small enough to be plausibly dependant on imports. Furthermore, we assume no starting point for wood and paper use in the time period covered here, as wood has been a primary resource for centuries. Prior to 1961, we therefore back-casted wood and paper production using population growth rates until 1820.

We classified paper and paperboard production and trade from FAO as semi-finished products (p3) and additionally added UN Comtrade data on trade in final products such as books or printed media (p4). We additionally excluded secondary paper production from reported total paper production and trade as explained in detail in Section 7. In line with paper production, we classified solid-wood production and trade flows reported by FAO as semi-finished products (p3) and additionally added UN Comtrade data for trade in final wood products such as furniture (p4).

Industrial roundwood (after bark removal) is usually classified as the raw product (p2) for wood and paper semi-finished products [42] and its production and trade is also comprehensively reported by the FAO in volume measures, which we as well converted to weight measures using IPCC factors [63]. In this unique case, we could therefore compile production data on two stages of the processing chain and could derive losses that occur from the processing of industrial roundwood to wood semi-finished products (stemming from water loss, energy recovery of waste products, unrecoverable sawdust, etc.) by mass-balancing of the data. We derived processing loss rates by calculating industrial roundwood (IRW) consumption (production + imports – exports) and then dividing the difference to total production of wood semis (sawnwood, wood-based panels, paper, others) by IRW consumption. For many countries and years, IRW consumption seems to be vastly underestimated as the amount of reported semis production is higher than the reported IRW consumption. In accordance with the literature [3,66,76,103], we assume accounts of wood semi-finished products to be more comprehensively reported and developed an approach to estimate IRW production where IRW data is inconsistent, as described in the following paragraph.

From a comprehensive global study on wood flows conducted by Bais et al. [3], we could derive plausible global processing losses for sawnwood, paper and other products for the years 1990, 2000 and 2010 by dividing the reported unrecovered wastes by respective IRW inputs. The resulting factors were interpolated for the years between and held constant for earlier and later years and weighted by production flows of the specific product group to derive global average processing loss factors. The resulting global average processing losses do not vary much over time, ranging from 32% in 1900 to 31% in 2016. We then used global average processing losses to extrapolate IRW consumption from data on the production of semi-finished wood products for datapoints where the calculated losses are below a third of the global average (11%). In addition, a few countries do not report production of semi-finished products but still IRW consumption. For these cases, we assumed IRW consumption to be zero to comply with the rule of prioritizing reported production of semi-finished products. The global average processing losses we derived from this approach then range from 36% in 1900 to 33% in 2016, which is in line with the literature [3,71,84]. In total, we end up with 31% of datapoints for IRW production to be estimated as explained above, but total mass of estimated datapoints only accounts for 3% of the total mass of IRW production from 1900 to 2016. Uncertainties of these estimation procedures are assessed as explained below in 10.

### 6.5 Iron & steel

Iron and steel have been the most abundantly used metal resource throughout human history and the reporting of iron and steel flows has been well-established for a long time. Steel is an alloy with the major component being iron, with typically a few percent of carbon to improve its fracture resistance and strength compared to iron and small concentrations of other metals to attain certain properties (which are also accounted for in this category). Global steel production took off with industrial production around 1860, before iron products have either been wrought or cast [98].

There are several international data sources on iron and steel production and trade flows (e.g [17,121,140]), which have all been utilized by various studies over the last decades (e.g [46,57,97,98]; [129]). For this database, we build on the approach of Pauliuk et al. [97], who comprehensively compiled international and selected national data sources to quantify iron and steel flows for all countries worldwide from 1700 to 2008. We updated this time series until 2016 using data from the World Steel Association [140] and Comtrade [114]. The Steel Statistical Yearbooks from worldsteel entail detailed information on pig iron, directly reduced iron (DRI) and crude steel production and trade back to the year 2000 [140].

We compiled data on production of crude steel and casting iron from Pauliuk et al. [97] and worldsteel (2020). Cast iron has been calculated by using a global ratio of cast iron to pig iron and DRI consumption (after trade). Pauliuk et al. [97] estimated this share to decrease from 100% in 1860 to 7% in 2008, which we then held constant for the years until 2016. As Pauliuk et al. [97] mostly derived their data from worldsteel data for recent years and worldsteel shows a higher country coverage, we derived production of raw products from worldsteel accounts for the years 2000–2016 and used data from Pauliuk et al. [97] before 2000. Prior to 1900, Pauliuk et al. [97] only published their final GAS estimates publicly, which we therefore used as a proxy for production flows and assumed trade as well as processing losses in following processing steps to be zero. To derive iron and steel trade flows for all processing stages, we applied the approach proposed by Pauliuk et al. [97] to Comtrade data, which goes back to 1962. Earlier trade flows back to 1900 have been estimated; trade data handling is described in detail in Section 5. We additionally excluded production and trade of secondary iron and steel from the accounts as described in Section 7. The novel accounts for primary iron and steel production agree well with production accounts on pig iron reported by USGS and BGS.

We classified crude steel and casting iron as raw products (p2) and additionally added UN Comtrade data on trade in raw products (p2), semi-finished products like steel plates or rails (p3) and final products like machinery or appliances (p4).

### 6.6 Aluminium, copper, zinc, lead, chromium, manganese, nickel and tin

Non-ferrous metals account for only a small fraction of total metal consumption of countries compared to iron and steel, although they are of huge importance to industrial production and their environmental impacts are wide-ranging [55]. In this database, we include the most widely used and technologically crucial metals aluminium, copper, lead, zinc, chromium, manganese, nickel and tin, as they cover the majority of non-ferrous metals used throughout history in terms of mass [54,107]. Their applications and properties are wide-ranging [127]. Aluminium is largely used in transport utilities, packaging, building and construction and electricity-related uses and its use increased vastly over the 20th century due to its relatively low costs, high conductivity, light weight and durability. Copper is used as a conductor of heat and electricity and for many other purposes for a long time, as it is one of the few metals that can occur in nature in a directly usable metallic form. Lead was used extensively due to its abundance, low extraction costs and beneficial properties until the discovery of its toxicity in the late 19th century led to the phase-out of its use in many applications. However, it is still used in several applications that are supposed to not affect human health. Zinc, chromium, nickel and tin are largely used in alloys, mostly to enhance the properties of steel.

Many organisations and research groups investigate extraction, production and use processes of these metals and data reporting has also already a long history. Two major primary data reporters on historical flows throughout the 20th century are the Br. Geol. Surv. [5], [6], [7], [8], [9], [10], [11], [12], [13], [14], [15], [16], [17] and the United States Geological Survey [121]. BGS reports production as well as imports and exports of a wide range of mineral commodities and represents, as far as this is possible, official national accounts. In the case of metals, production data of different steps in metal processing can be selected (mine production, smelter, refining), each expressed in terms of metal content. Data are reported for all countries in the world and can be downloaded for the years from 1970 onwards. The years prior to 1970 are available as pdfs in BGS archives, dating back till 1913. The USGS database contains – besides comparable country-level data – partly very detailed information on the structure of the mineral industry within specific countries, particularly in terms of commodity, major operating companies, major equity owners, location of main facilities, and annual capacity. This provides valuable information, especially on metal contents or primary and secondary production. The time period accessible via the online database usually dates back to 1990, earlier publications until the 1930s are only available as pdf files.

Often databases do not refer to standard statistical codes, therefore some caution is required when working with more than one database to avoid either incomprehensive or double counting. We therefore decided to gather primarily data from one of the two data sources and use the other for cross-checking. We also compared flows from the BGS and the USGS database and data was highly coinciding. As the BGS database provided easily accessible data for 20 more years, we decided to primarily use BGS data and digitalized information for early years (1913–1970) from the pdf yearbooks (1925, 1927, 1930, 1933, 1936, 1939, 1948, 1953, 1959, 1965, 1971, 1973) using optical character recognition (OCR) software. For aluminium and copper, we additionally used data from the World Bureau of Metal Statistics [131], who provide a comprehensive production dataset for major metals from 1950 onwards, providing a higher level of completeness than BGS.

For the purpose of gathering data on raw products (p2), we compiled data on production and trade of primary aluminium, refined copper, refined lead, zinc slabs, refined nickel, refined tin, chromium ore and concentrates and manganese ore. Details on the categories included in the production and trade data compiled from BGS and WBMS can be found in Table 7. Categories in trade data from 1913 to 1970 are highly diverse, which is why we only show some of the main selected categories. For chromium and manganese, BGS only reports data on ore extraction. To estimate the metal content in gross ore accounts, we combined ore grades for chromium and manganese from different sources to derive regional and temporal differences [18,73,121]. We multiplied these ore grades with production and trade of ores and concentrates, but of course not with trade in metals themselves. Comparing the novel metal accounts with estimates from Wang et al. [127] at that stage delivers high overlaps on the global level, which is why we are confident about the initial production estimates.

**Table 7:** Categories included in production and trade statistics from BGS (and WBMS for aluminium and copper).

Metal	Production (BGS & WBMS)	Trade 1970–2016 (BGS & WBMS)	Trade 1913–1970 (BGS)
**Aluminium**	Primary aluminium	Unwrought, unwrought alloys	Unwrought, semi-manufactures, alloys, …
**Copper**	Refined copper	Unwrought, unwrought refined, unwrought alloys	Rough and refined copper, semi-manufactures, alloys, unwrought, …
**Lead**	Refined lead	Unwrought, unwrought refined, unwrought alloys, semi-manufactures	Crude and refined unwrought, alloys, semi-manufactures, …
**Zinc**	Zinc (slab)	Unwrought, unwrought alloys, crude, refined	Refined zinc, semi-manufactures, unwrought, …
**Chromium**	Chromium ore and concentrates	Ores and concentrates, metal	Metal and alloys, chromate and bichromate, …
**Manganese**	Manganese ore	Ores and concentrates, metal	Concentrates, metal, alloys, …
**Nickel**	Nickel (smelter/refinery)	Unwrought, unwrought alloys	Unwrought, alloys, semi-manufactures, …
**Tin**	Tin (smelter)	Unwrought, unwrought refined, unwrought alloys, semi-manufactures	Unwrought, alloys, semi-manufactures, …

Comparing BGS trade datasets with Comtrade data for the years 1962–2016 indicates a higher completeness of Comtrade data (after modification as explained in Section 5). BGS trade data is unfortunately not giving a common classification of traded products, as countries report their trade at various aggregation levels (which we depict in the uncertainty estimates). To be sure to avoid either double counting or underrating of data for at least the years after 1962, we only used trade data from Comtrade, which is following a common classification system, for the metals for which comprehensive datasets could be derived: aluminium, copper, lead and zinc. For these metals, we added BGS data for the years prior 1962 for processing stage 2, which connects well to Comtrade estimates. For chromium, manganese, nickel and tin, only BGS data was available and used for the first processing step, trade at following processing stages was assumed zero. BGS trade data for many developing countries has not been available for recent years after 2002 due to unknown reasons, which is why we extrapolated data for these years, so that global exports more or less equal global imports.

Further data processing and estimation steps have been conducted as described in Section 3. For the metals, for which only total production (primary and secondary) was reported (copper, lead, zinc, nickel) and for trade flows of all metals, we excluded secondary production as described in detail in Section 7. Back-casting of production flows prior to 1913 has been deliberated for each material separately. The first large-scale production method for aluminium was developed in 1886, after which production increased rapidly as prices fell drastically [36]. We therefore introduced a technological starting point for aluminium production at 1886 until which we linearly reduced production towards zero, in case the last datapoint was higher than 5 kt/yr. The use of copper, lead, zinc and tin intensified in line with industrialization processes [73], which is why we back-casted their production flows with GDP changes rates, in case the last datapoint in 1913 was higher than 5 kt/yr for copper and zinc and higher than 2 kt/yr for lead, nickel and tin. As chromium and manganese have almost entirely been used as components of steel alloys, we back-casted their production flows with global crude steel production change rates back to 1820. Back-casting of trade flows was done using growth rates of global trade flows (constant US$) sourced from Federico and Tena-Junguito (2016) until 1900, before we assumed trade flows to be negligible.

The novel database constitutes a comprehensive quantification of all major stock-building material flows at different production stages. Therefore, an important issue is to avoid double-counting of materials that have been combined in material compounds or alloys. These considerations largely matter for metals, which exist and are reported in various forms of alloys. While it is beyond the scope of this paper to investigate alloys of all other metals, we at least consider major iron and steel alloys, which cover the bulk use of metals in society. Some of the metals we quantified in this work are to a large extent used as components of (mostly stainless) steel: chromium at about 90%, manganese at around 85% and nickel at around 68% of their total production flows. Data on end-uses for several years and some developed countries indicating metallurgical use could be derived from USGS and WBMS yearbooks ([119,[130], [132]). As these fractions are already accounted for in the total amount of crude steel production, we subtracted them from the accounts on the production of other metals in processing stage 2 to avoid double-counting in the final consumption estimates. All other compound usages of metals (e.g. zinc galvanizing) have not been considered in this database and are continuously reported in their elementary form.

As we could not compile full trade datasets for chromium, manganese, nickel and tin and the estimates for these metals are in total not very robust, we decided not to show these results separately, but included them into the aggregate of all other metals. While this group represents an approximate account for the mass flows of all other metals, the data quality is in general too low to warrant a more detailed analysis.

### 6.7 Plastics

The category plastics includes a wide range of synthetic and semi-synthetic materials that usually use polymers as main ingredients. Modern plastics are produced industrially and are mostly derived from fossil fuel-based petrochemicals in processes of polymerization, copolymerization, condensation, polycondensation and polyaddition [77]. Plastics production and especially trade flows are hard to estimate, as plastics are included in a huge variety of raw, semi-finished and final products. We utilized international databases ([61,^116^,[117], [118]) and various additional data sources (e.g [2,39,99]) to compile country-level plastics production and trade flows. The final dataset covered seemingly complete time series for 127 countries from 1970 to 2016.

Plastics production data can be primarily found in the United Nations Industrial Commodity Production Statistics database (UNICPS) [116,117]. Data has been combined from two different UNICPS datasets from 1970 to 2003 and 1995–2016 and for the latter dataset, if no weight measure is available, conversions of values in US$ to kg using average UNICPS kg/US$ factors have been conducted. For the early dataset, no additional monetary or physical data was available for conversion. Details on which commodity categories have been compiled and which conversion factors have been applied can be found in Table 8.

**Table 8:** UNICPS plastics commodities used and conversion factors applied.

UNICPS dataset 1970–2003		UNICPS dataset 1995 - 2016		
UNICPS code	UNICPS commodity category	UNICPS code	UNICPS commodity category	kg/US$
351310	Alkyd resins	34710–1	Polyethylene having a specific gravity of less than 0.94, in primary forms	0,95
351313	Aminoplastic resins	34710–2	Polyethylene having a specific gravity of 0.94 or more, in primary forms	0,87
351316	Phenolic and cresylic plastics	34720–1	Polystyrene, in primary forms	0,78
351318	Artificial resins and plastic materials	34720–2	Styrene-acrylonitrile and acrylonitrile-butadiene-styrene copolymers, in primary forms	0,52
351319	Polyethylene	34730–1	Polyvinyl chloride, in primary forms	0,99
351320	Ethylene-vinyl acetate copolymers	34740–1	Polycarbonates, in primary forms	0,47
351322	Polypropylene	34740–2	Polyethylene terephthalate, in primary forms	0,99
351323	Acrylic polymers	34790–1	Polypropylene, in primary forms	3,52
351326	Polyacetals	34790–2	Acrylic polymers in primary forms	1,00
351328	Polyvinyl chloride	34790–3	Polyamides in primary forms	0,51
		34790–4	Amino-resins, phenolic resins and polyurethanes, in primary forms	1,42
		34790–5	Silicones in primary forms	0,29
		34800–0	Synthetic rubber	0,19

However, UNICPS data coverage on plastics production was still very poor, reporting fragmented data only for 82 countries of the sample. We therefore additionally sourced data on plastics feedstocks, i.e. fossil fuel products serving as inputs to plastics production processes, from the World Energy Balance database of the International Energy Agency [61] and the UN energy statistics [118]. Plastics feedstocks from IEA were compiled by summing up non-energy uses of the chemical and petrochemical industry for ethane, liquified petroleum gases (LPG), Naphtha, natural gas liquids (NGL) and other oil products. The 2017 IEA dataset only contains data from 1960/1971 to 2015, we therefore had to estimate 2016 values using average growth rates of 2011–2015. Plastic feedstocks from UN energy statistics were compiled by summing up non-energy use of liquified petroleum gases (LPG), Naphtha, natural gas liquids (NGL) and other oil products. From these two datasets, we build a comprehensive dataset on plastic feedstocks based on data availability, covering 118 countries from 1970 to 2016.

To account for losses occurring during processing of plastics feedstocks to plastic raw products, we applied a global yield factor of 86% to all datapoints in the plastic feedstock dataset. We derived this average yield factor by calculating input-output-ratios of plastic feedstocks to plastic raw products by combining process efficiencies from steam cracking and polymerization processes [77] and weighted these by 2015 global production shares of plastics raw products [47]. Thereafter, we combined this dataset with several time series from UNICPS and some additional data on national plastics production [2,39,64,99,100,106,111].

Back-casting of plastics production was either based on growth rates of historic oil production [101], if available, or based on GDP. While the use of natural rubber was well established by the start of the twentieth century, the major growth period of the plastics industry was from 1930 onwards in certain countries. Mass production then took off in the 1940s and 1950s [138]. We therefore decreased the share of plastics in oil production, trade and GDP till zero in 1950 for most countries (if the last available datapoint was above 100 kt/yr) and for some frontrunners till zero in 1930 (France, Germany, Italy, Japan, UK, USA, Russia).

We classify plastics production from polymerization, etc. as raw products (p2). Trade flows of plastics could be plausibly identified and classified as flows of raw, semi-finished, final and waste products using material contents of UN Comtrade data as described in Section 5.

### 6.8 Container and flat glass

Glass is a transparent, non-crystalline material that is mostly produced from silica, the primary constituent of sand. It is widely used for applicative, technological and decorative purposes. In this database we include flat glass (architectural and window glass) and container glass (bottles, jars, drinkware and bowls). We exclude other glass products (fibres, tableware, technical glass, …) as they only account for a small percentage of total glass production and data availability is severely limited on these specific products. Trade flows of flat and container glass were compiled from UN Comtrade data as described in Section 5, but could not be discerned into different glass products.

Flat and container glass production accounts, which we classify as raw products (p2), can be found in the United Nations Industrial Commodity Production Statistics database (UNICPS) [116,117]. Data has been combined from two different UNICPS datasets from 1950 to 2003 and 1995–2016 and conversions have been applied to all datapoints where no weight measure was available but other physical or monetary units. The conversion factors applied could either be derived from calculating average factors over all countries and years from UNICPS or Comtrade data or – if this approach could not deliver plausible results – from additional sources. Details on which commodities have been compiled from UNICPS, how they have been distinguished into flat and container glass and which conversion factors have been applied (incl. their sources) can be found in Table 9. Although several conversions were conducted, UNICPS data coverage was still very poor, with only reporting fragmented data on 78 countries for flat glass and 75 countries for container glass. Global and world region estimates do not come close to previous estimates of Krausmann et al. [74] or [137] and national data often appeared implausible. Therefore, we required additional data sources to be able to develop plausible estimates on glass production.

**Table 9:** UNICPS glass commodities used and conversion factors (incl. sources) applied. All kg/$ factors have been derived from the UN Comtrade database (see Section 5).

Category	UNICPS commodity category	Conversion factors				Sources		
		kg/u.	kg/m^2^	kg/m^3^	kg/$	kg/unit	kg/m^2^	kg/m^3^
**1995–2016**								
**Flat glass**	Slivers, rovings, yarn and chopped strands, of glass	no physical values available	0.36					
**Flat glass**	Voiles, webs, mats and other articles of glass fibers except woven fabrics	1005	5.3	2580	0.36	UNICPS	UNICPS	[109]
**Flat glass**	Safety glass	494	25	2510	0.38	UNICPS	[41]	[60]
**Container glass**	Bottles, jars and other containers (except ampoules), stoppers, lids and other closures, of glass	0.50	–	969	1.54	[125]		[90]
**Flat glass**	Drawn glass and blown glass, in sheets	0.09	14	833	0.39	[40]	UNICPS	[41]
**1950–2003**								
**Flat glass**	Glass, drawn or blown, in rectangles, unworked	0.09	10	833	2.47	[40]	UNICPS	[41]
**Flat glass**	Glass, cast, rolled, drawn or blown	0.09	9.7	833	2.3	[40]	UNICPS	[41]
**Flat glass**	Glass fibres (including glass wool)	1005	5.3	2580	0.45	UNICPS	UNICPS	[109]
**Flat glass**	Toughened or laminated safety glass	494	8.9	2510	0.48	UNICPS	UNICPS	[60]
**Container glass**	Glass bottles and other containers of common glass	0.7	-	969	2.3	UNICPS		[90]

In addition to open access data sources, we purchased additional data on global national-level production capacities and trade data, both distinguished into different glass products from the glass industry network glassglobal [51]. National production capacities for 2017 were available for 49 countries for flat glass and 93 countries for container glass and agreed well with previous estimates on global and national glass production [21,22,50,74,85,134]. We estimated global production of flat and container glass on the basis of data provided by UNSD [116] and industry statistics [49,83,94], which was already compiled by Krausmann et al. [74]. Country-level estimates were then derived using national glassglobal [51] production capacity shares for 2017 and temporal changes in production between world regions from 1900 to 2015 derived from Wiedenhofer et al. [137]. UNICPS data was then only included for a few countries where no production capacities but trade data was available.

Trade data from glassglobal was based on the more detailed HS commodity classification and therefore only dating back to 1988, while Comtrade data was based on the SITC1 classification and available since 1962. Due to the longer time period provided by Comtrade and general high overlaps of the two data sources, we chose Comtrade data as the primary source on glass trade data and used glassglobal trade data to derive shares of flat or container glass of total national glass trade from 1988 to 2016 (with shares held constant for earlier years). We then used these shares to distinguish trade in glass and glassware from Comtrade into the two categories. For earlier years, estimation based on monetary world trade growth rates were derived, as described in more detail above. For container glass, we additionally excluded production and trade of secondary materials as described in Section 7. Back-casting of glass production flows prior to 1900 was based on population trends, with t/cap of glass production remaining constant.

We classified flat and container glass production as raw products (p2). Trade flows of flat and container glass could be identified and classified as flows of raw (p2) and semi-finished (p3) products. Unfortunately, no data is available on trade of glass in final products used as packaging container material (p4).

## 7 Secondary vs. primary production

Secondary resources that are recovered from waste flows are used in increasing quantities, as both waste flows and recycling rates increase especially in industrialized regions [56]. However, this study focuses on the quantification of all primary materials extracted and processed into stock-building products. The quantification of end-of-life flows, which would be needed to systematically and consistently quantify the amount of secondary resources, is beyond the scope of this study. Therefore, we decided to exclude secondary materials from all total production and trade flows (including primary and secondary materials) from the data and analysis presented in this paper.

Production flows of paper, iron and steel, copper, lead, zinc and container glass were only reported in totals, i.e., primary and secondary materials could not be distinguished. Approaches to exclude secondary materials have been chosen individually for each material and will be explained in Section 7.1. To stay consistent with production accounts, we also corrected trade flows for their secondary material content for paper, iron and steel, aluminium, copper, lead, zinc and container glass, as further explained in Section 7.2. We assume that recovered materials included in trade flows of concrete, asphalt, bricks and other metals are negligible. For wood, plastics and flat glass, we assume that the amount of recovered materials in production as well as trade flows are negligible, which is substantiated by the scientific literature [3,47,134].

Production flows have been corrected based on national level data, while trade flows have been corrected using annual global averages. Global average factors for both production and trade flows are given in Table 10.

**Table 10:** Shares of primary materials in total production (primary and secondary) applied to material flows reported in totals.

*Material*	*Global average of raw production primary shares* (weighted by national production flows)		*Trade primary share of world markets* (weighted by raw product export flows)	
	1900	2016	1900	2016
**Concrete**	Data for primary materials available		100%	100%
**Asphalt**	Data for primary materials available		100%	100%
**Bricks**	Data for primary materials available		100%	100%
**Paper**	78%	59%	82%	62%
**Wood**	Data for primary materials available		100%	100%
**Iron & steel**	80%	64%	80%	61%
**Aluminium**	Data for primary materials available		100%	78%
**Copper**	83%	80%	87%	87%
**Lead**	59%	46%	79%	46%
**Zinc**	55%	92%	86%	97%
**Other metals**	Data for primary materials available		100%	100%
**Plastics**	Data for primary materials available		100%	100%
**Container glass**	100%	86%	100%	82%
**Flat glass**	Data for primary materials available		100%	100%

### 7.1 Excluding secondary materials from production flows

To derive country-level primary production shares for the relevant materials, we developed several approaches and used a variety of data sources. For paper, we compiled data on recovered paper from the FAO [42]. As recovered paper is not fully materially recycled, we know that only about 81% of recovered paper flows are used as secondary materials [123]. For several countries and years, secondary materials exceeded total production compiled as described in Section 6.4. We therefore replaced data points when primary shares drop below 10% by interpolation or the world average (see Table 10), as a certain share of primary materials is likely being used [122,123].

Iron and steel production and trade accounts also include large shares of secondary materials, as the recycling of iron-based metals has a long tradition [1,102]. As there is no comprehensive international dataset available that distinguishes between primary and secondary production of iron and steel, we decided to base the herein used assumptions on main production technologies dominating iron and steel production. Steel is nowadays basically produced either in blown oxygen converters (BOF), which typically use 80% primary materials and 20% secondary materials, or in electrical furnaces (EF), which typically use 20% primary materials and 80% secondary materials [105]. We therefore compiled data from the World Steel Association (worldsteel) on crude steel production from BOF and EF from 2000 until 2016, that together account for 99.65% of total steel production in 2016, and calculated primary production shares by weighting the technology-determined input factors by the amounts produced either in BOF or EF. If no data on BOF/EF production was available, we used a constant primary share of 80%, as BOF has been the predominant production technology worldwide [105].

**Fig. 2:**
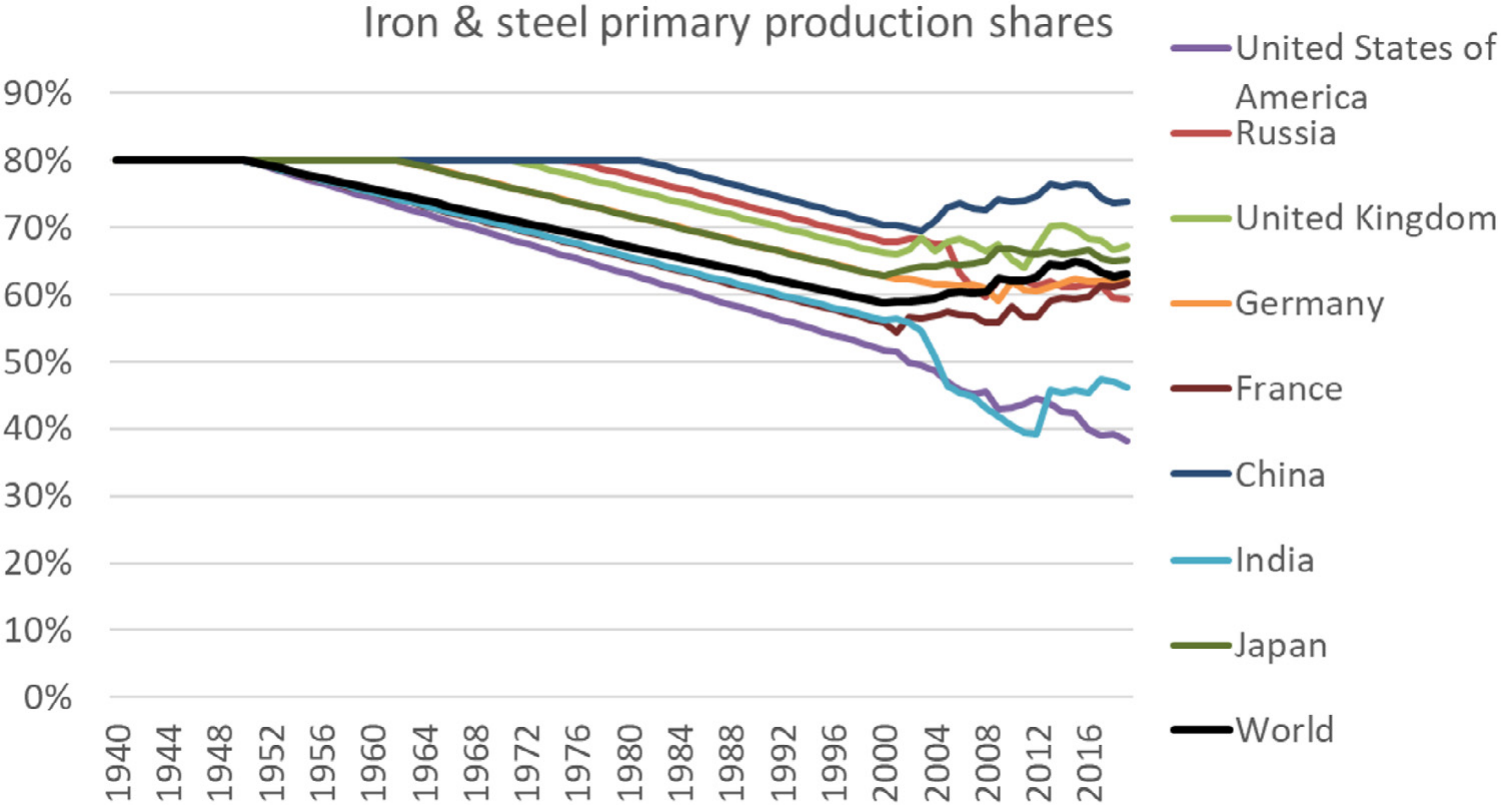
Primary production shares for iron and steel production for the world and some selected countries from 1940 to 2018.

The wide-scale use of EF for steel production actually took off in the 1950s and delivered 26% of global production 2016; before 1950, only BOF and related technologies have been used [82]. Before worldsteel data was available, we therefore back-casted global average primary shares to linearly increase from 59% in 2000 to 80% in 1950 and back-casted country-level shares using these global growth rates. If countries didn't reach 80% primary share by 1950 using global growth rates, we linearly interpolated between 1950 and 2000, so that all countries end up at a primary share of 80% in 1950. World average primary shares and primary shares of some selected countries are shown in Fig. 2.

For aluminium, we compiled data on primary production from WBMS and BGS and therefore only had to exclude secondary production from trade data using global average trade primary shares (as explained below). We could access data on secondary production of aluminium from the WBMS yearbooks for the years 2000–2019 ([130], [132]), from which we could derive primary shares of total production and the global average trade shares. Mass-recycling of aluminium started to be applied wide-scale only in the 1940s [78] and we therefore assumed a global trade primary share of 100% before 1940 and linearly interpolated between 75% primary share in 2000 and 100% in 1940.

**Fig. 3:**
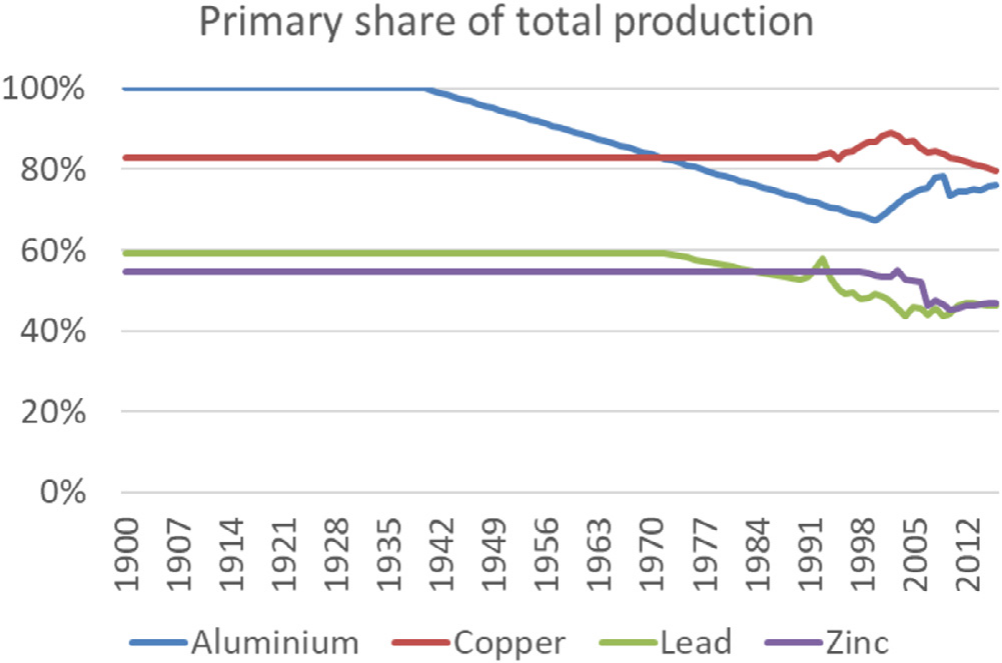
Global average primary production shares for aluminium, copper, lead and zinc from 1900 to 2016.

Copper, lead and zinc production is only reported in total production by the BGS back until 1913 (which we used as main data source), but the USGS provides data on primary and secondary production back until 1990 [121], from which we could derive country-level primary production shares for the three metals. For countries where no data on secondary production was available, we used the global average production share (see Fig. 3). Before 1990, we extrapolated primary shares of total production by holding values constant. Global average raw production shares of aluminium, copper, lead and zinc derived from USGS and WBMS data are shown in Fig. 3. As all other metals account for only a very small fraction of all materials investigated in this database and data compilation is in general not complete, we did not correct for secondary production and trade flows for these metals.

For container glass, we could hardly find information on the amount of recovered glass reused as production input. Therefore, we derived some conservative assumptions based on the scientific literature to exclude secondary production from container glass production and trade flows. We distinguished countries into countries with higher or lower income based on the World Bank classification for 2016 [139]. For higher income countries, we assumed a production primary share of 77% based on recycling shares reported for the USA [38]. For lower income countries, we assumed a minimum recycled share of 5% (and a respective primary share of 95%) that is commonly reported by glass industries [21,22,51,134]. We assumed the recycling of container glass to really take off around 1960 in industrialized regions and around 2000 in developing regions [21], and therefore back-casted primary shares in 2016 by linear interpolation to 100% in 1960 for higher income countries and to 100% in 2000 for lower income countries.

### 7.2 Excluding secondary materials from trade flows

In the previous section, we explained how we derived the shares of primary materials in total production for each material individually. For trade flows, we then applied a consistent and transparent approach to all materials equally by weighing these shares of primary materials in production (PrimShare) by the amounts of exports in raw materials (EXP_p2) for each country (cr), as shown in the following equation:

(1) $$PrimShare_{trade}\;=\;\sum_{i=1}^{n}\frac{\left[EXP\_p2_{cr1}*PrimShare_{cr1}\;+\;EXP\_p2_{cr2}*PrimShare_{cr2}\;+\;\ldots \;+\;EXP\_p2_{crn}*PrimShare_{crn}\right]}{EXP\_p2_{tot}}$$

We could therefore adjust global primary production shares by countries’ involvement in the world market for raw products and correct for the material composition of domestically used products. We assume that excluded secondary materials include recovered end-of-life waste as well as recovered processing wastes and that the final datasets are therefore fully compatible with the MFA scheme presented in Section 1.

## 8 Processing wastes

From primary inputs we furthermore deduct losses and wastes occurring during the different processing stages to identify the fraction of extracted primary materials that goes into manufacturing and construction. Unrecoverable processing wastes (*waste_unrec*) or losses represent the unrecoverable part of processing wastes that is treated further by waste management or dissipatedly lost to the environment. Recoverable processing wastes (*waste_rec*) represent the recoverable part of occurring wastes and are especially for metals often also designated as new scrap. Recoverable waste flows can, but do not necessarily have to be recycled. In this study, we do not consider recycling processes and therefore do not quantify the amount of materials actually recovered.

We conducted an in-depth literature review to gather information on these two parameters on the national level and based the chosen approach on findings from the literature. Detailed information on processing wastes and losses does not seem to be well established in ew-MFA literature yet, especially when it comes to non-metallic materials. To apply a consistent approach for all materials and countries in the database, we decided to derive global factors for each material based on either global studies or national-level studies, if no global studies where available. We then applied these global shares to all countries during the whole time-period from 1900 to 2016.

Table 11 gives an overview on processing waste factors from the literature for production of semi-finished (P3) and final products (P4) and derived global factors and assumptions used in this study (written in bold). Production output of raw products is derived directly from primary data sources (see Section 3) and we estimate wastes occurring during the processing of raw materials to raw products (P2) using global factors as described in the following Section. For all materials, the herein assumed processing losses are slightly higher than what the authors assumed in previous studies [71,74,137].

**Table 11:** Processing waste factors of total production for each material and their literature sources. We distinguished recoverable (*waste_rec*) and unrecoverable waste rates (*waste_unrec*). Global factors are applied for all countries and held constant over time.

Material	Region/ Country	Process parameters	(3) Semi-finished products	(4) Final products	Literature sources
**Concrete**	China	waste_unrec	–	1.4%	[23,81]
	India	waste_unrec	–	3.2%	[86]
	Japan	waste_unrec	–	1.5%	[69]
	South Korea	waste_unrec	–	2.0%	[69,95]
	Sweden	waste_unrec	–	10.0%	(S [128].)
	Brazil	waste_unrec	–	19.8%	[19,104]
	Netherlands	waste_unrec	–	3.0%	[19]
	USA	waste_unrec	–	3.5%	[19,32]
	WORLD	waste_unrec	**–**	**5.5%**	average of the above
**Asphalt**	WORLD	waste_unrec	**–**	**3%**	own assumption
**Bricks**	USA	waste_unrec	–	4.0%	[32]
	South Korea	waste_unrec	–	3.0%	[69]
	China	waste_unrec	–	7.0%	[81]
	India	waste_unrec	–	5.7%	[86]
	Brazil	waste_unrec	–	17.0%	[19]
	UK	waste_unrec	–	5.0%	[33]
	Netherlands	waste_unrec	–	6.0%	[19]
	WORLD	waste_unrec	**–**	**6.8%**	average of the above
**Paper**	country-specific	waste_unrec	**balance IRW & semis**	**–**	[3,42]
	WORLD	waste_rec	**–**	**5.0%**	[122]
**Wood**	country-specific	waste_unrec	**balance IRW & semis**	**–**	[3,42]
	WORLD	waste_rec	**–**	**17.5%**	[3]
**Iron & steel**	WORLD	waste_unrec	**1.0%**	**–**	[98]
	WORLD	waste_rec	**9.0%**	**14.2%**	[98]
**Aluminium**	WORLD	waste_unrec	**–**	**–**	[4]
	WORLD	waste_rec	**30.9%**	**15.2%**	[4]
**Copper**	WORLD	waste_unrec	**0.5%**	**0.5%**	[52]
	WORLD	waste_rec	**1.9%**	**18.0%**	[59,141,142]
**Lead**	WORLD	waste_unrec	**–**	**–**	[59]
	WORLD	waste_rec	**6.0%**	**–**	[59]
**Zinc**	WORLD	waste_unrec	**0.7%**	**1.5%**	[88]
	WORLD	waste_rec	**10.8%**	**13.3%**	[88]
**Other metals**	WORLD	waste_unrec	**0.7%**	**1.0%**	average of factors for metals above
	WORLD	waste_rec	**11.7%**	**15.2%**	average of factors for metals above
**Plastics**	China	waste_rec	–	6.4%	[64]
	Europe	waste_rec	0.6%	6.7%	[67]
	Netherlands	waste_rec	2.5%	7.8%	[65]
	Austria	waste_rec	2.5%	7.5%	[124]
	WORLD	waste_rec	**1.9%**	**7.1%**	average of the above
**Container glass**	WORLD	waste_unrec	**–**	**1.5%**	own assumption
**Flat glass**	Japan	waste_unrec	–	1.0%	[69]
	South Korea	waste_unrec	–	2.0%	[69]
	WORLD	waste_unrec	**–**	**1.5%**	average of the above

For construction materials, many literature sources analyze losses that occur directly on construction sites (e.g [19,95]). As we assume for some of the materials (concrete, bricks, flat glass) that they are entirely used for construction, we can consider these loss rates as unrecoverable wastes at the latest processing stage (construction of final products). As we are not yet able to distinguish between different end-uses of materials (e.g. buildings, consumer goods, transport, …), we cannot identify the shares lost during construction for materials, which are not exclusively used for construction purposes, from this literature. This especially applies to wood, iron & steel, aluminium, copper and plastics, for which we therefore neglected construction site losses and waste flows are very likely underestimated.

Metal processing is optimized to avoid unrecoverable wastes, which is why most of the waste is termed recoverable in the literature and often no information on unrecoverable waste is available [59]. That is also the case for plastics, where losses only occur in the primary production processes of primary plastics from raw materials [77,124]. We therefore assume all wastes during further processing of plastics to be recoverable. Losses from processing of industrial roundwood to wood and paper are calculated based on the difference between reported or estimated data for industrial roundwood and wood and paper production as described in Section 6.4. For asphalt as well as for container glass, we could not find any literature sources quantifying wastes occurring during production processes. As it is very unlikely that no wastes would occur, we assume for asphalt half of the construction site losses of concrete (as asphalt is easier to reuse) and for container glass the same share of unrecoverable cullet as for flat glass.

## 9 Quantification of the extraction of raw materials on the global level

For this database, we could mainly compile exogenous input data from international databases for production and trade of raw and further processed products. It is not in the focus of this paper to also quantify extraction and trade flows of raw materials on the national level, as it is commonly done in ew-MFA [45]. We therefore only estimate raw material extraction on the global level using factors to account for the difference between production of raw products and the extraction of raw materials required for this. This difference can also be seen as processing wastes as defined in Section 8. Processing wastes at this stage comprise, for example, tailings from the processing of gross ore to metals, CO_2_ emissions from the calcination of limestone, bark from the processing of roundwood, or water vapor from changes in moisture content of clay during brick production [73]. For non-metallic minerals and other materials, wastes at this stage are not as high as for metals, but still not negligible. It is beyond the scope of this study to distinguish recoverable and unrecoverable wastes from raw material processing, we therefore assume that recovery of these material flows is not economically feasible and therefore assume them to be unrecoverable.

Coefficients to extrapolate the required raw material extraction from production of raw products (kg/kg) and their sources are given in Table 12. We applied these coefficients to global production of raw products (P2) assumed them to be constant over time (with the exception of metal ore grades). For construction materials and glass, losses occur during cement and bricks production as CO_2_ emissions due to the calcination of limestone and the moisture loss during drying of clay [74] For metals, their ore grades, i.e. the relation between metal content and gross ore, allow for the estimation of gross ore content from pure metal content. Ore grades are highly variable across ores, mines and time, which is why we gathered global ore grades from USGS [120] for each metal separately and considered their variation over the whole time period from 1900 to 2016.

**Table 12:** Coefficients for the conversion of raw products to their extracted materials (kg/kg). Factors are applied for all countries and held constant over time (except for metals where we consider temporal variations in ore grades, values given here are for 2016). If more than one raw material is used for the production of a raw product, conversion factors are provided for all individuals (left) as well as the sum (right) for all material components.

Raw material	*raw product-to-raw material*		Raw product	Sources
Limestone	*1.25*	*1.45*	Cement	[74]
Clay	*0.20*			
Crude oil	*1.00*		Bitumen	–
Clay	*1.35*		Bricks	[74]
Ind. roundwood overbark	*1.13*		Ind. roundwood underbark	[63,115]
Iron ore	*1.89*		Iron & steel	[120]
Bauxite	*2.26*		Aluminium	[120]
Copper ore	*95.24*		Copper	[120]
Lead ore	*16.95*		Lead	[120]
Zinc ore	*10.21*		Zinc	[120]
Other metal ores	*80.65*		Other metals	[120]
Crude oil	*0.83*	*1.17*	Plastics	[47,77]
Natural gas	*0.34*			
Industrial sand	*0.73*	*1.12*	Container/Flat glass	[74]
Soda ash	*0.22*			
Limestone, dolomite and other	*0.17*			

For plastics, we derived information on processing losses from primary inputs to primary plastics from two main global studies [47,77] as described in the specific Section 6.7 and assumed 29%/71% of the required raw materials to be natural gas/crude oil [77]. In the case of wood, wood removals are usually reported under bark, i.e. without bark, in forestry statistics, although wood is removed including bark and a significant fraction of the bark is even subject to further socio-economic use (e.g. energy production) [73]. We therefore estimate wood removals including bark to be 1.13 times the amount of industrial roundwood without bark [63,115].

## 10 Assessing data robustness and uncertainty

Data availability and quality to quantify material flows on a national level is often problematic due to data scarcity, differing definitions and coverages across sources and fragmentary data reporting especially for historical periods. In this research, we paid special attention to address these challenges of data compilation in a transparent way and developed a comprehensive assessment of uncertainties for each input datapoint as well as parameters. We decided to score the reliability of data sources and estimation methods based on an evaluation framework proposed by Laner et al. [75] and translating these to normally distributed standard deviations for each datapoint. The assessment methods for input datasets and parameters are explained in detail below in Sections 10.1 - 10.4.

In addition, we surveyed how many of the datapoints have been estimated in comparison to the datapoints coming from data sources in terms of counts and in terms of percentages of total mass. We also calculated for each dataset how much of the total mass (tons) has been estimated as outlined above for each year. Results for this evaluation are given in detail in Section 10.5. The total sum of the accumulated error derived from mass-balancing corrections (explained in Step 10 in Section 3) is presented in Section 10.6.

### 10.1 Framework to systematically score data quality and assess uncertainty

For the quantification of potential deviations of the reported data, we harnessed and adapted a systematic approach of data quality assessment developed by Laner et al. [75], which is based on the so-called Pedigree matrix [58,133]. The matrix gives a comprehensive scoring of available datapoints along five independent data quality indicators: reliability, completeness, temporal correlation, geographical correlation and further technological correlation. *Reliability* relates to the primary data source and comprises an assessment of the reported data compilation and verification methods documented by the responsible institution or team. *Completeness* indicates if we assume possible over- or underestimations of the mass flow due to e.g. fragmentary reporting or potential double-counting. The indicators *temporal and geographical correlation* describe deviations in time and space from the actual date of interest. *Other correlation* takes other deviations into account such as conversion issues. Based on an evaluation of the data quality with respect to each indicator, corresponding scores (1–4) are derived. Differences in the compilation of statistical data (e.g. reporting standards of countries, …) of primary sources go beyond the scope of this assessment and are not reflected by this method.

In addition to the evaluation of data from sources, indicators for the evaluation of expert estimates are introduced, in case no published dataset or measurements could be accessed. The quality of expert estimates is based on the transparency and consistency of generating the estimate and the knowledge of the expert about the respective matter. An evaluation based on reliability, completeness, temporal, geographical and other correlation does not apply in that case and uncertainty values for the scores are defined separately. An overview of the evaluation criteria specified for each indicator is shown in Table 13.

**Table 13:** Qualitative evaluation criteria for the application of scores 1 to 4 on data quality indicators (adaption based on [75]).

Data quality criteria	Data quality scores			
	1	2	3	4
**Data reliability (R)**	Official topical databases, curated by expert organizations and validated through professional expertise (science, practitioners). *Examples*–IEA, Cembureau, FAO	Databases collecting information as provided, without harmonization or curation. Primary data sources are clear and documentation on issues is available; some level of quality control is applied. *Example*–Comtrade	Databases only containing fragmentary data, due to weak statistical collection and patchy primary data; primary sources unclear. *Example*–UNICPS	Database with some data, where methodology and primary data collection unclear. Potentially based on expert judgements.
**Completeness (C)**	All relevant flows included; indicator definitions are ident	Indicator definitions slightly different, but quantitatively main flows covered	Certainty of data gaps, very likely approximated	Only fragmented data
**Temporal correlation (T)**	Exact same time period	Deviation 1–5 years	Deviation 5–10 years	Deviation >10 years
**Geographical correlation (G)**	Studied region	Similar socio-economic region	Socio-economically slightly different region	Very different region
**Other correlation (O)**	No use of conversion factors necessary (besides simple unit transformations e.g. GJ to kWh, pound to gram)	Conversion between physical units or by using well-established conversion factors based on evidence from natural sciences (e.g. volume-to-weight)	Conversions via prices/from monetary data, conversion factors based on statistical evidence	Speculative conversions/ correlations between materials
**Expert judgement (EX)**	Judgement based on empirical data, fully informed	Structured expert estimate with some empirical data	Strongly generalized empirical data or verified information	Educated guess based on speculative assumptions

### 10.2 Systematically operationalizing the framework

Based on these definitions, we created a list of baseline scores for all main data sources we used in this database (Table 14), which we took as basic uncertainty for the datapoints we could directly use from these sources. In addition, we decided on individually deviating scores for data manipulations and estimations we performed to adjust and complete the datasets. If any estimation method has been applied for a certain datapoint, we exchanged the baseline scores of the database by the new scoring and could therefore individually assess the uncertainties of the herein used estimation methods in relation to each other. Here, we also introduced the expert judgement scoring, which suits best for many estimation procedures we applied. Details on the individual scoring of the estimation methods used can be found in Table 15.

**Table 14:** Baseline scoring (1–4) of data quality indicators for all major data sources used (adaption based on [75]). R–reliability, C–completeness, T–temporal correlation, G–geographical correlation, O–other correlation.

Data source	Material	Data quality score				
		*R*	*C*	*T*	*G*	*O*
IEA	Asphalt	*1*	*1*	*1*	*1*	*2*
IEA	Plastics	*1*	*2*	*1*	*1*	*3*
UNSD energy stat.	Plastics	*1*	*2*	*1*	*1*	*3*
EUROMAP	Plastics	*3*	*2*	*1*	*1*	*1*
UNICPS	Plastics	*3*	*2*	*1*	*1*	*1*
UNICPS	Asphalt	*2*	*2*	*1*	*1*	*2*
UNICPS	Bricks, glass	*3*	*3*	*1*	*1*	*3*
Cembureau	Cement	*1*	*1*	*1*	*1*	*2*
FAO	Wood	*1*	*1*	*1*	*1*	*2*
FAO	Paper	*1*	*1*	*1*	*1*	*1*
Comtrade	All materials	*2*	*3*	*1*	*1*	*3*
WSA	Steel	*1*	*2*	*1*	*1*	*1*
Pauliuk et al. (2013)	Steel	*1*	*2*	*1*	*1*	*2*
BGS	Metals	*1*	*2*	*1*	*1*	*1*
BGS	Chromium, manganese	*1*	*2*	*1*	*1*	*3*
WBMS	Aluminium, copper	*1*	*1*	*1*	*1*	*1*

**Table 15:** Individual uncertainty scoring of estimation procedures applied in the database. Here we see which estimation procedure was used for which materials and how they have been scored according to the different data quality indicators (R–Reliability, C–Completeness, T–Temporal correlation, G–Geographical correlation, O–Other correlation, EX–Expert judgement). T scores are given as defined in Table 13 according to the distance to the next real datapoint (dep.=depends). Details on estimation procedures can be found in the respective material section above.

Nr	Estimation procedure	Applied to:	Indicators	Scoring
**1**	Disaggregation by share of earliest datapoint in material flow of larger political aggregate	All materials	G; T	*2; dep.*
**2**	Additional data included or sums used	All materials	G	*1*
**3**	Equal distribution residual of larger political aggregate to countries without data	All materials	G	*3*
**4**	Interpolations (linear) & outlier correction	All materials	T	*dep.*
**5**	Future extrapolations using average growth of last 4 years (for max. 2 years)	All materials	EX	*2*
**6**	Future extrapolations holding last value constant (for more than 2 years)	Other metals	EX	*3*
**7**	Back-casting of trade data based on monetary world exports growth until 1900	All materials	EX	*4*
**8**	Back-casting of trade data based on growth rates of trade in semi-manufactures	Container/Flat glass	EX	*2*
**9**	Back-casting using Podobnik historic oil production growth rates	Bitumen, plastics	EX	*2*
**10**	Disaggregation (nr.1) based on back-casted estimates	All materials	EX	*4*
**11**	Back-casting of latest datapoint using technological starting point and GDP growth rates	Aluminium, plastics	EX	*3*
**12**	Back-casting of latest datapoint using GDP growth rates	Cement, asphalt, copper, zinc, lead	EX	*3*
**13**	Back-casting of latest datapoint with steel production growth	Other metals	EX	*3*
**14**	Back-casting of latest datapoint using population growth rates	Bricks, Wood, paper	EX	*3*
**15**	Back-casting based on population growth rate of world region	Wood	EX	*4*
**16**	Semi-finished production estimate based on global share of ind. roundwood production	Wood	C	*2*
**17**	Ind. roundwood estimate from semi-finished production using global average processing losses	Wood	C	*4*
**18**	Estimation based on per-capita material flow average of high/low income countries	Bricks	EX	*4*
**19**	Estimate based on average per-capita material flow of countries in the same GDP-decile	Bitumen	EX	*3*
**20**	Estimate based on world region developments from Wiedenhofer et al. [137] disaggregated with production capacities	Container/Flat glass	EX	*3*
**21**	Complementary data sourcing from official databases e.g. UNICPS	Bitumen, bricks, plastics, glass	See Table 14	
**22**	Complementary data sourcing from scientific studies	All materials	Individually assessed	
**23**	Sand and gravel estimate based on real data	Sand and gravel	EX	*2*
**24**	Sand and gravel estimate based on estimated data	Sand and gravel	EX	*3*

### 10.3 Translating data quality scores into quantitative uncertainty ranges

**Fig. 4:**
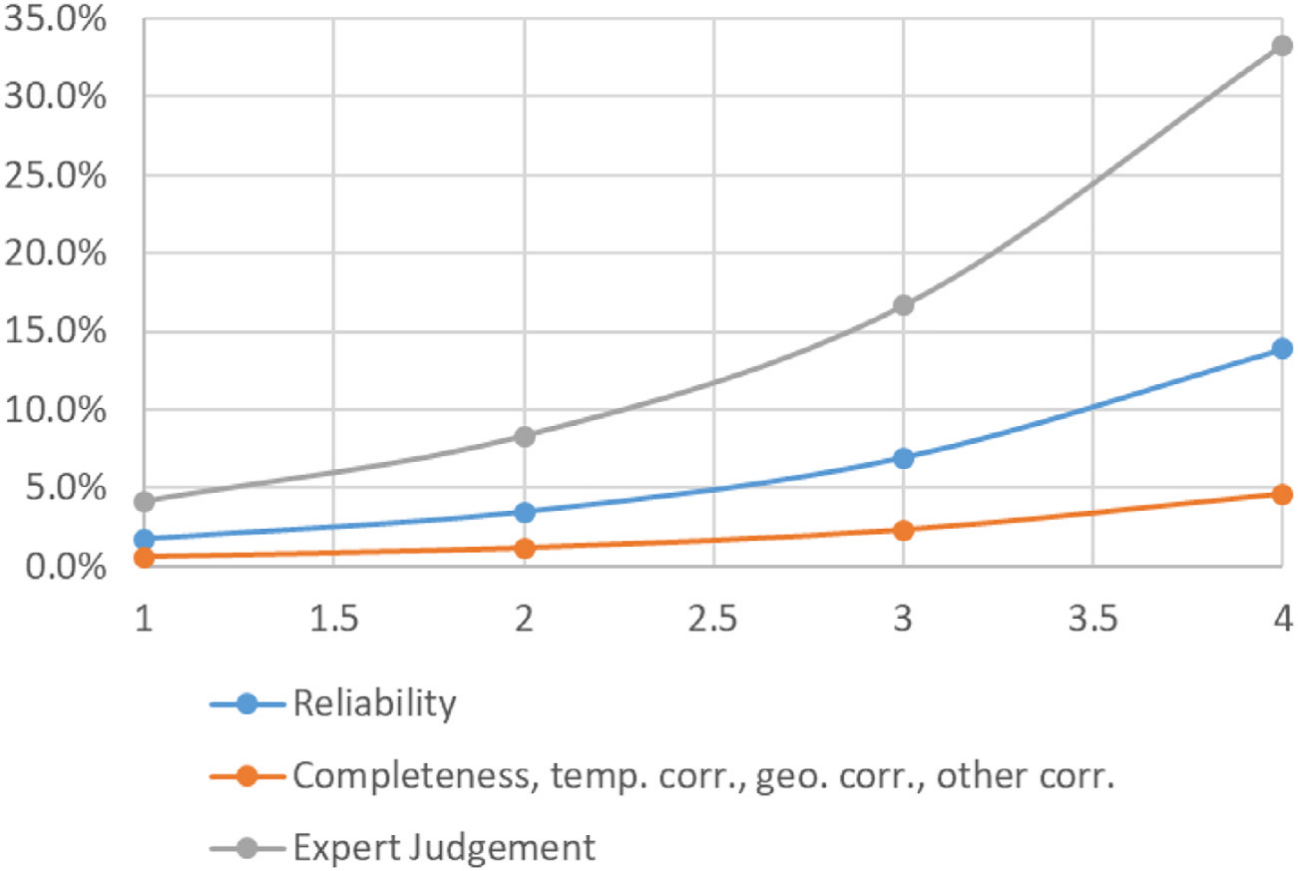
Functions used to translate ordinal scores (1–4) of data quality indicators into coefficients of variation (CV). The function given for completeness here also applies to temporal, geographical & other correlation.

In the next step, uncertainty scores were translated from the ordinal scale to a rational scale using following equations Eqs. (2)–((4), which are also displayed in Fig. 4. Deviations from the functions proposed by Laner et al. [75] were chosen as it was intended to not exceed maximum coefficients of variation (CV) of 33%, so that minimum error ranges could not turn data points into negative values, which could hinder subsequent uncertainty estimation methods. Furthermore, we chose to apply exponential functions and not linear functions, as an exponential increase in uncertainties seemed to be more likely. To fit the function, an exponential function was calculated that would result in CV_tot_ (see Eq. (5)) of 33.3% in case all data quality indicators would assume the worst quality level. The proportions of the worst quality score to all other scores were kept as suggested in Laner et al. [75].

(2) $$CV_{C,G,T,O}=0.00167*\;e^{1.105x}$$

(3) $$CV_{R}=0.00167*\;e^{1.105\left(x-1\right)}$$

(4) $$CV_{EX}=\;0.004*\;e^{1.1067x}$$

All uncertainty ranges are assumed to be normally distributed, given by mean (the actual datapoint) and standard deviation. The error range was expressed as a coefficient of variation (CV), i.e., the percentage of the respective parameter value containing 68% (or one standard deviation) of the stochastic realizations of these variables. Uncertainty estimates are derived for all material flows using the characterizing functions for each uncertainty criteria as shown in Fig. 4.

For estimated datapoints categorized as expert judgements, total CV for each datapoint is already given by Eq. (4). For data from sources, which has been evaluated along the five criteria reliability, completeness, temporal, geographical and other correlation, total CV is calculated using the following formula:

(5) $$CV_{tot}=\sqrt{CV_{R}^{2}+CV_{C}^{2}+CV_{G}^{2}+CV_{T}^{2}+CV_{O}^{2}}$$

The total error ranges given as coefficients of variations (CV) can then easily be translated into standard deviations by multiplying CVs by the actual datapoints and can then be further added up following Gaussian error propagation rules along the processing stages. Following calculated material flows then entail uncertainties of all previous flows and can then be used further by applying methods to derive total systematic uncertainties e.g. by applying Monte-Carlo simulations, which is beyond the scope of this work.

### 10.4 Presentation and validation of aggregated uncertainty ranges

**Fig. 5:**
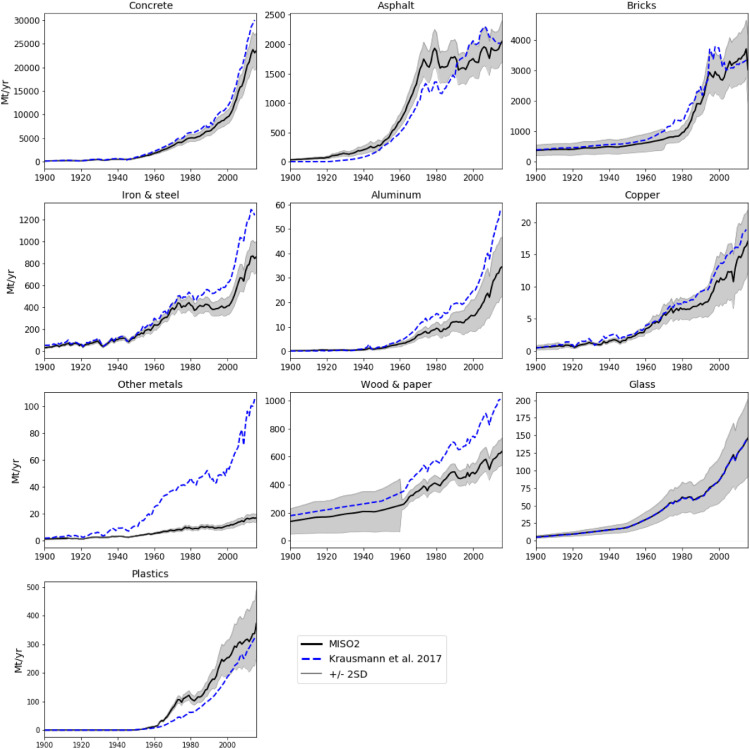
Comparison of global GAS_prim_ estimates in the herein presented database (incl. uncertainty ranges for 95% of the data (+/- 2SD)) with primary-inputs-to-stock estimates from [74].

**Fig. 6:**
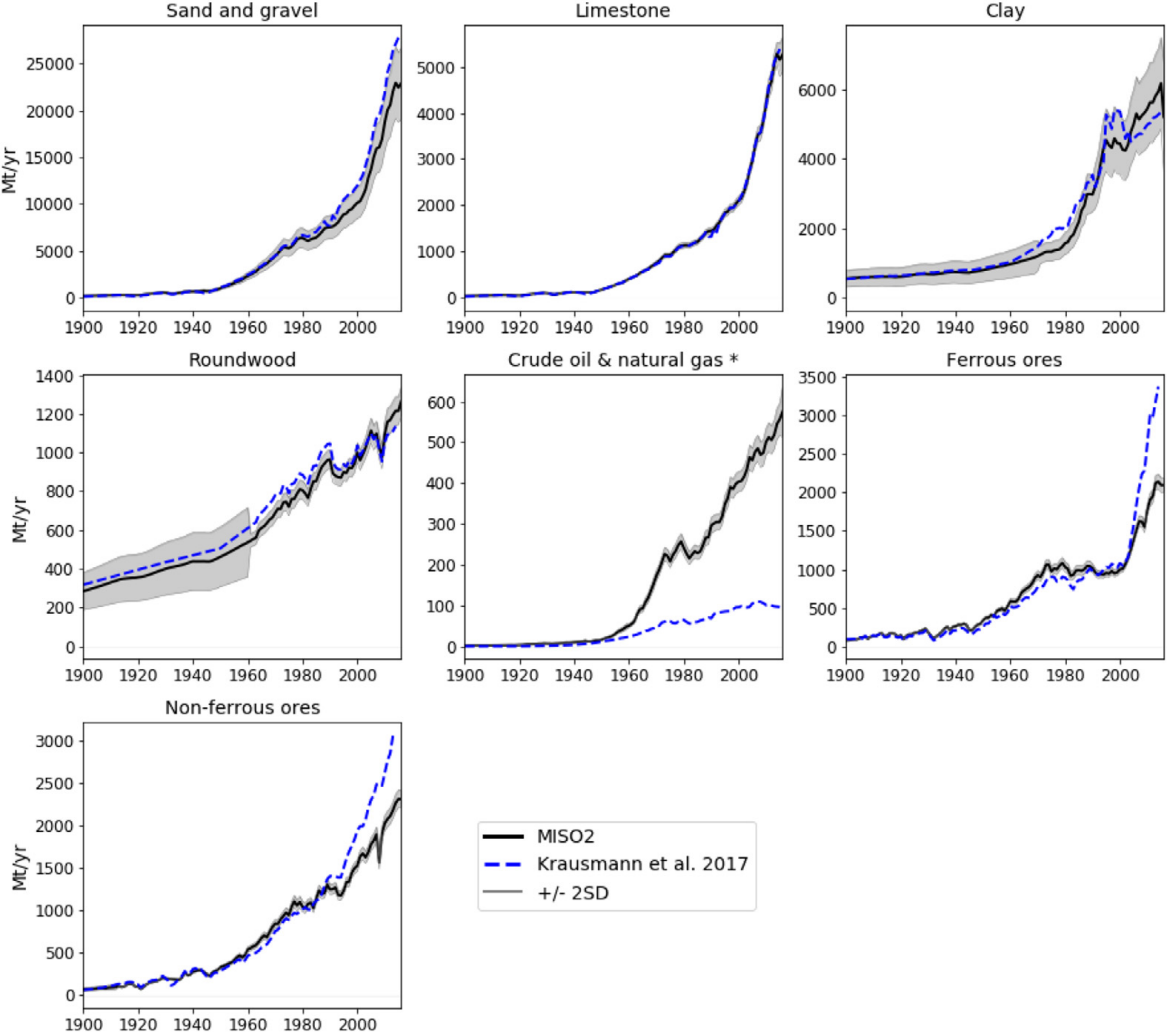
Comparison of global material extraction estimates in the herein presented database (incl. uncertainty ranges for 95% of the data (+/- 2SD)) with material extraction estimates from Krausmann et al. [74]. * crude oil & natural gas only from bitumen and plastics production.

In the following section, the results of the developed uncertainty assessment are presented for primary gross additions to stocks (GAS_prim_) and extraction of stock-building materials on the global level and compared to previous global top-down estimates from [72,74,136]. Mean estimates and uncertainties are given by ±2 standard deviations (SD, 95% of total distribution) for GAS_prim_ estimates in Fig. 5 and global stock-building material extraction estimates in Fig. 6, in comparison with primary inputs to stocks and global material extraction estimates from Krausmann et al. [74]. Material categories differ between extracted materials and final processed products.

**Fig. 7:**
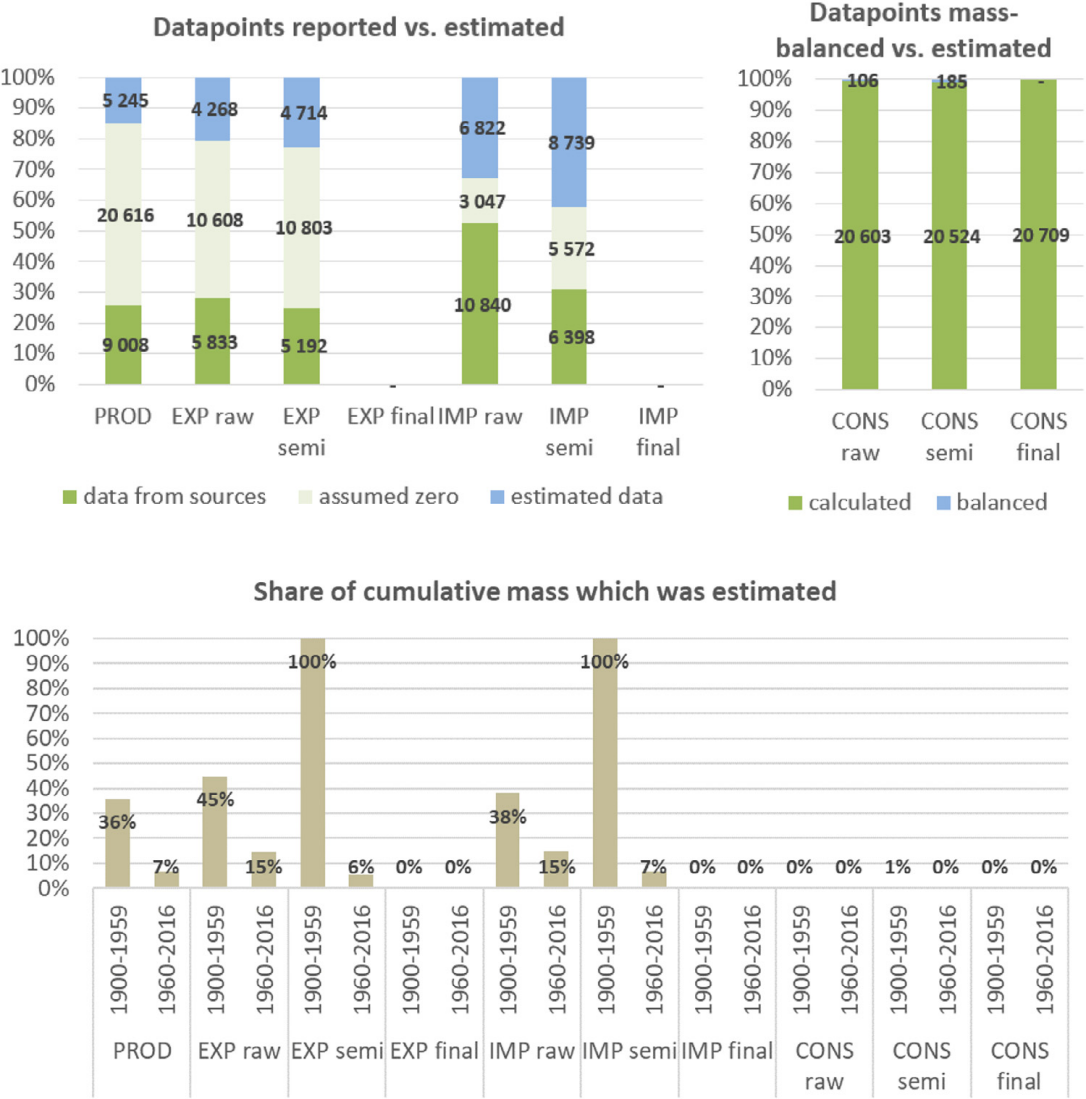
Counts on data from sources vs. estimated data for **cement**.

**Fig. 8:**
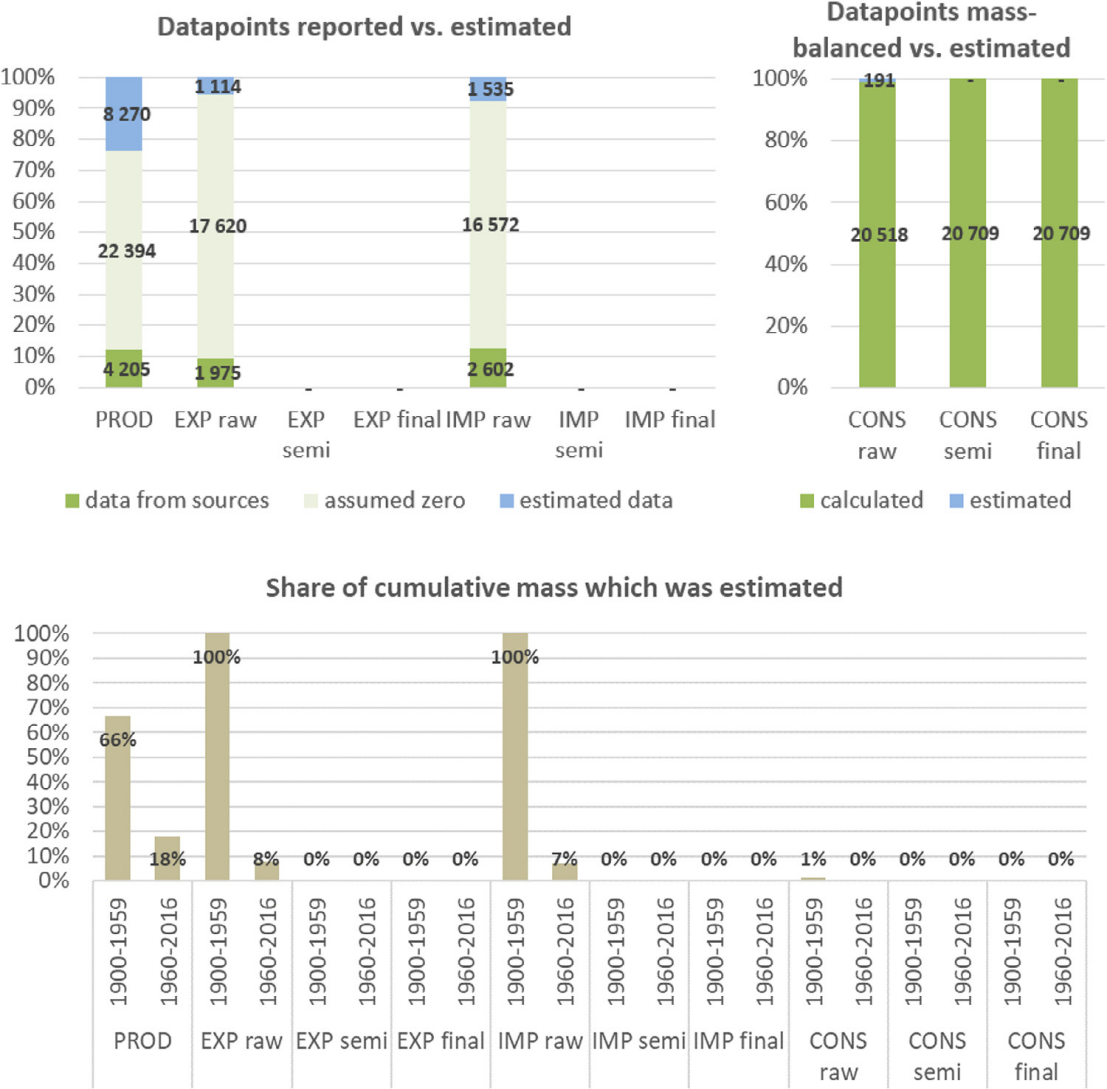
Counts on data from sources vs. estimated data for bitumen.

**Fig. 9:**
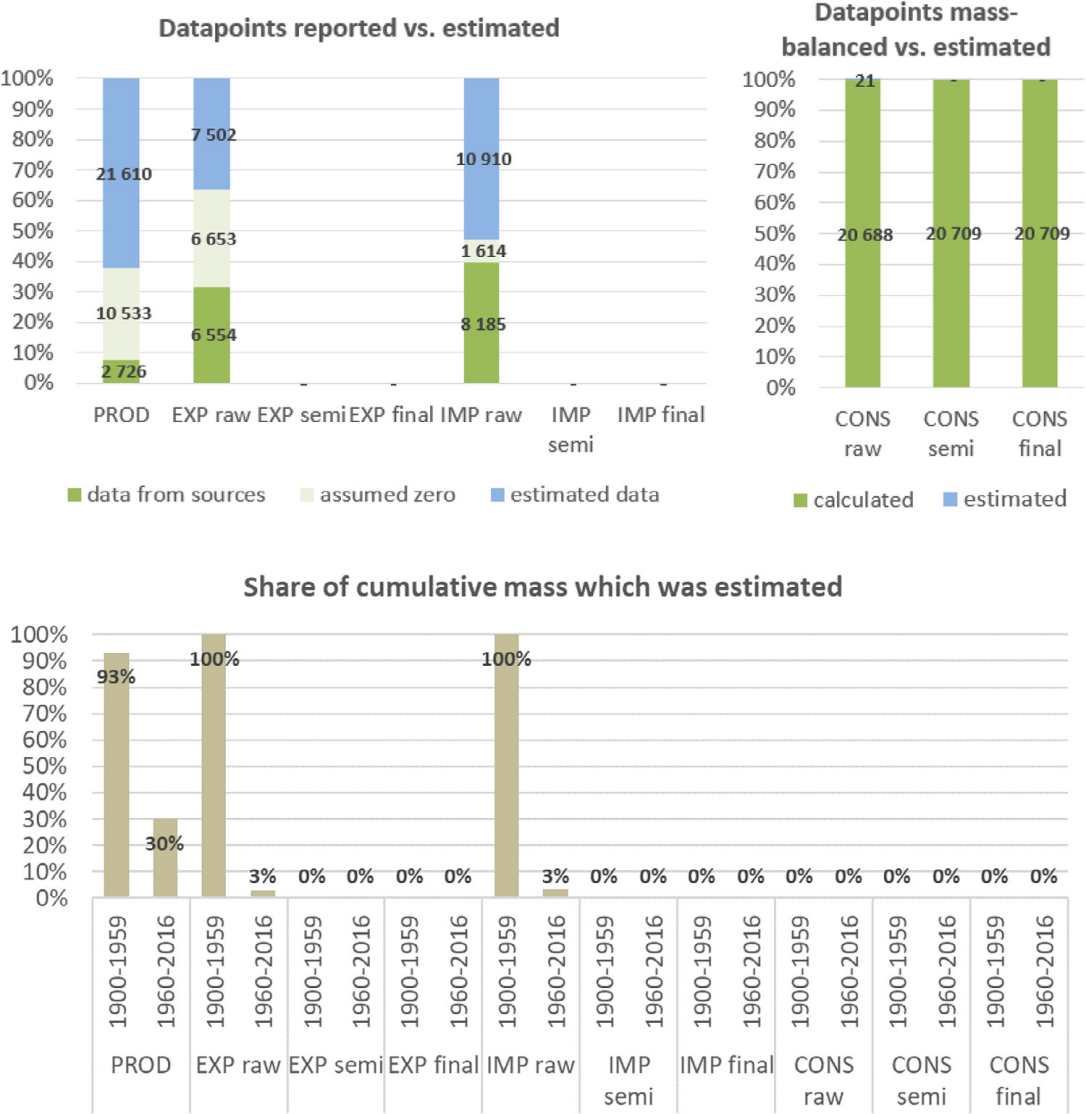
Counts on data from sources vs. estimated data for **bricks**.

**Fig. 10:**
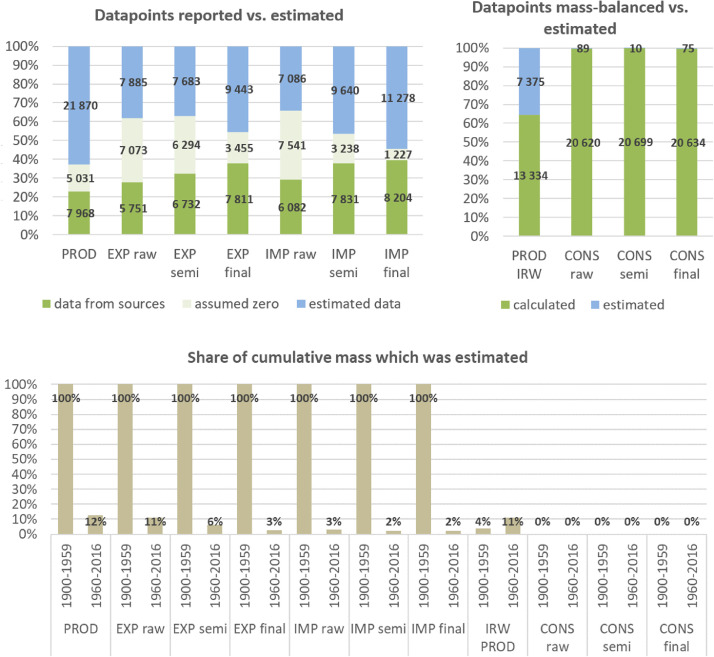
Counts on data from sources vs. estimated data for **wood**.

**Fig. 11:**
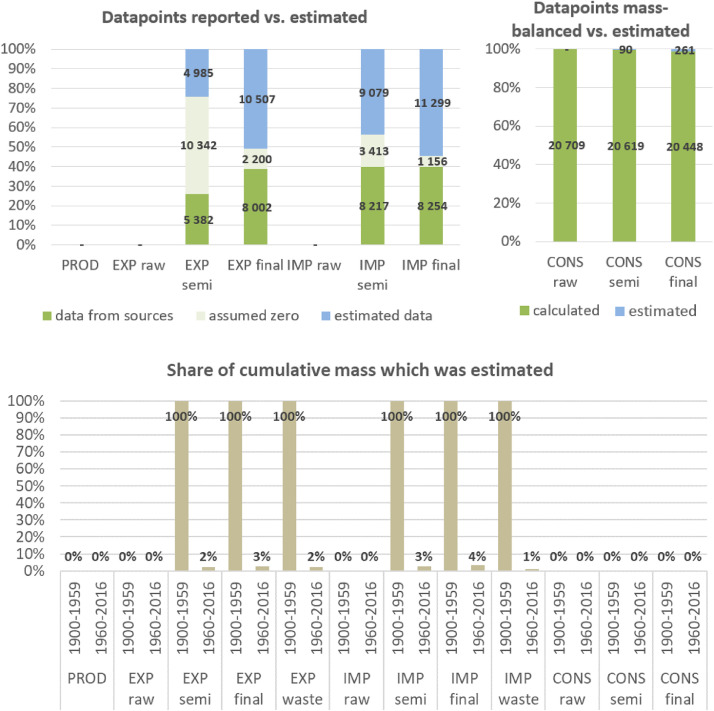
Counts on data from sources vs. estimated data for **paper**.

**Fig. 12:**
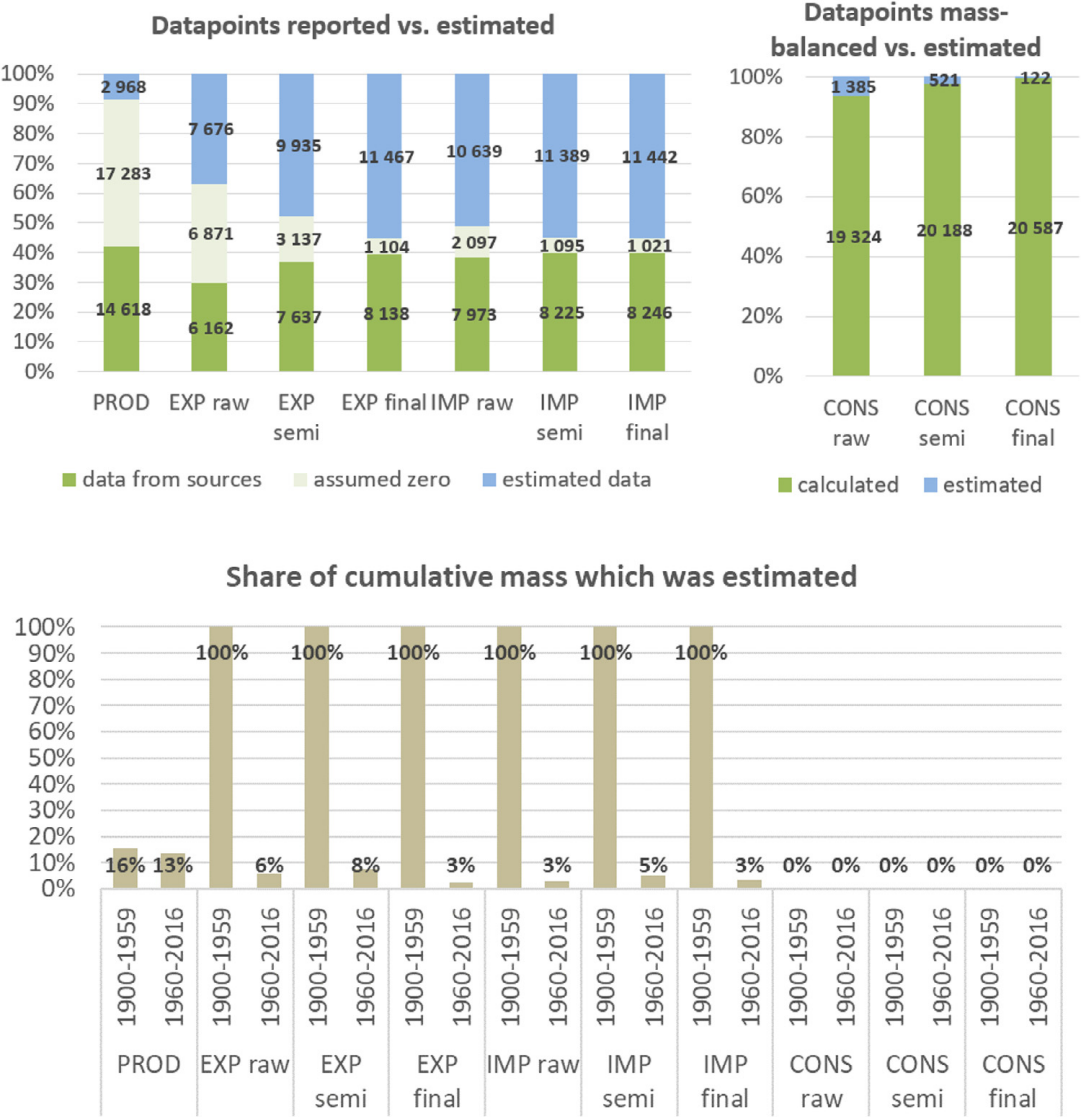
Counts on data from sources vs. estimated data for **iron and steel**.

**Fig. 13:**
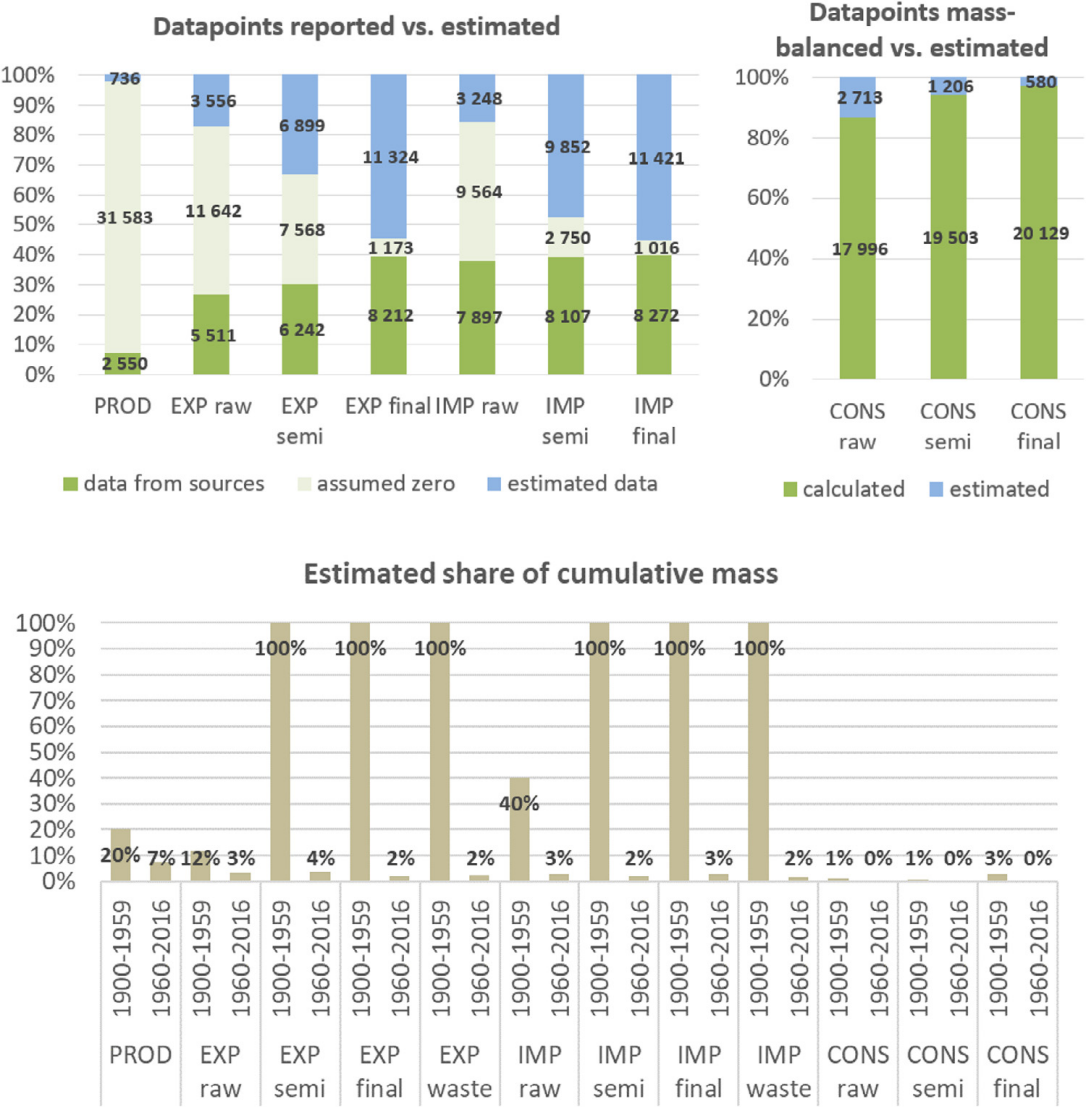
Counts on data from sources vs. estimated data for **aluminium**.

**Fig. 14:**
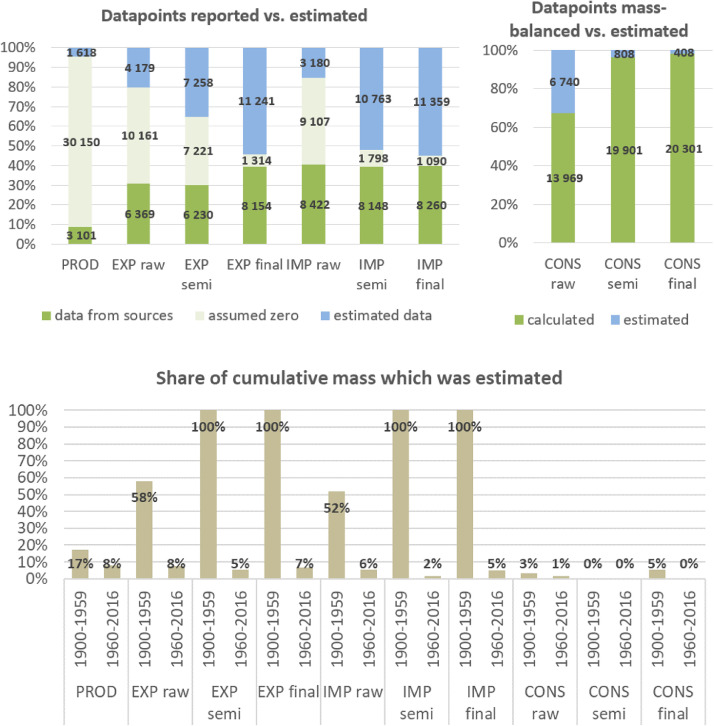
Counts on data from sources vs. estimated data for **copper**.

**Fig. 15:**
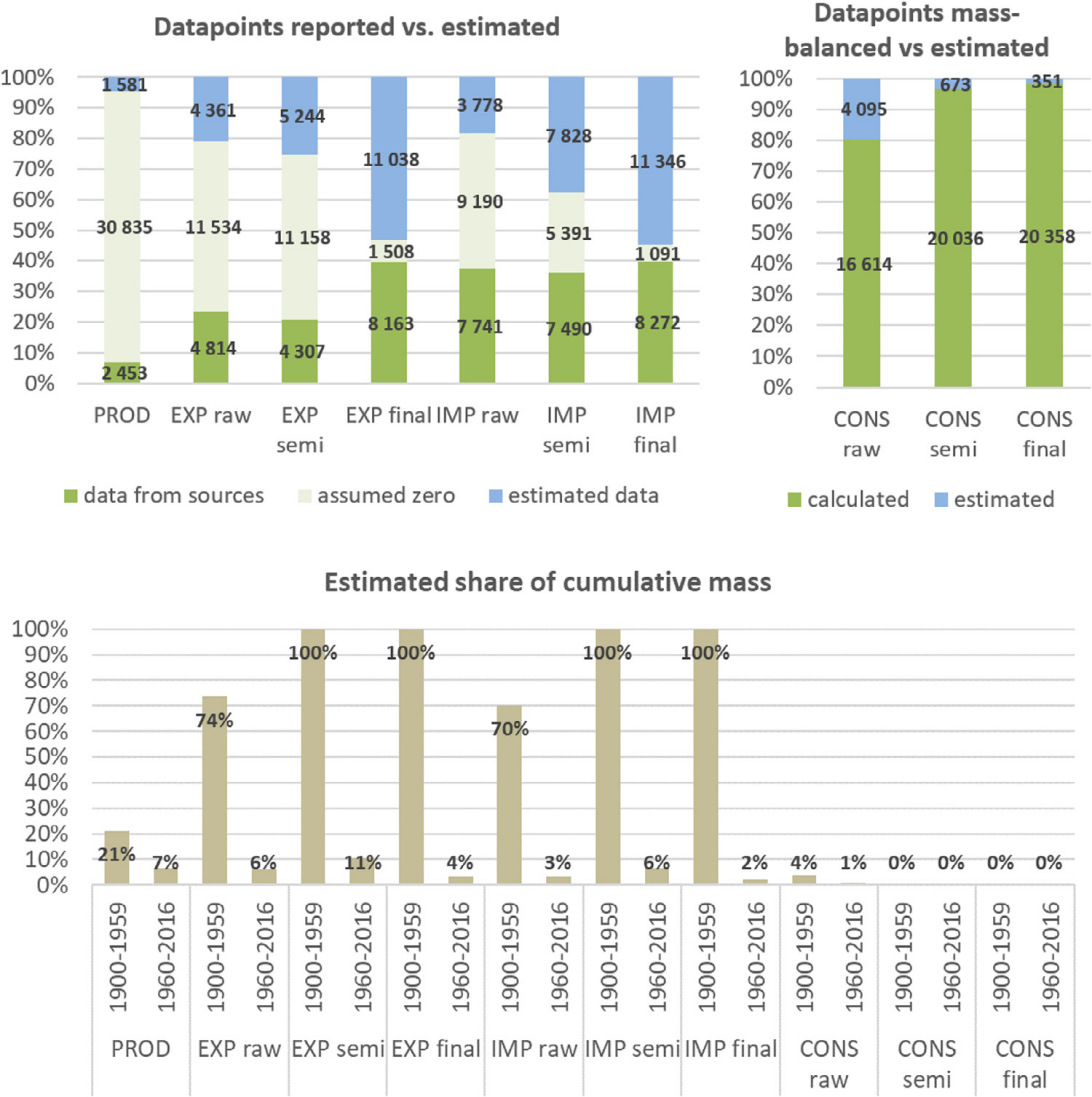
Counts on data from sources vs. estimated data for **zinc**.

**Fig. 16:**
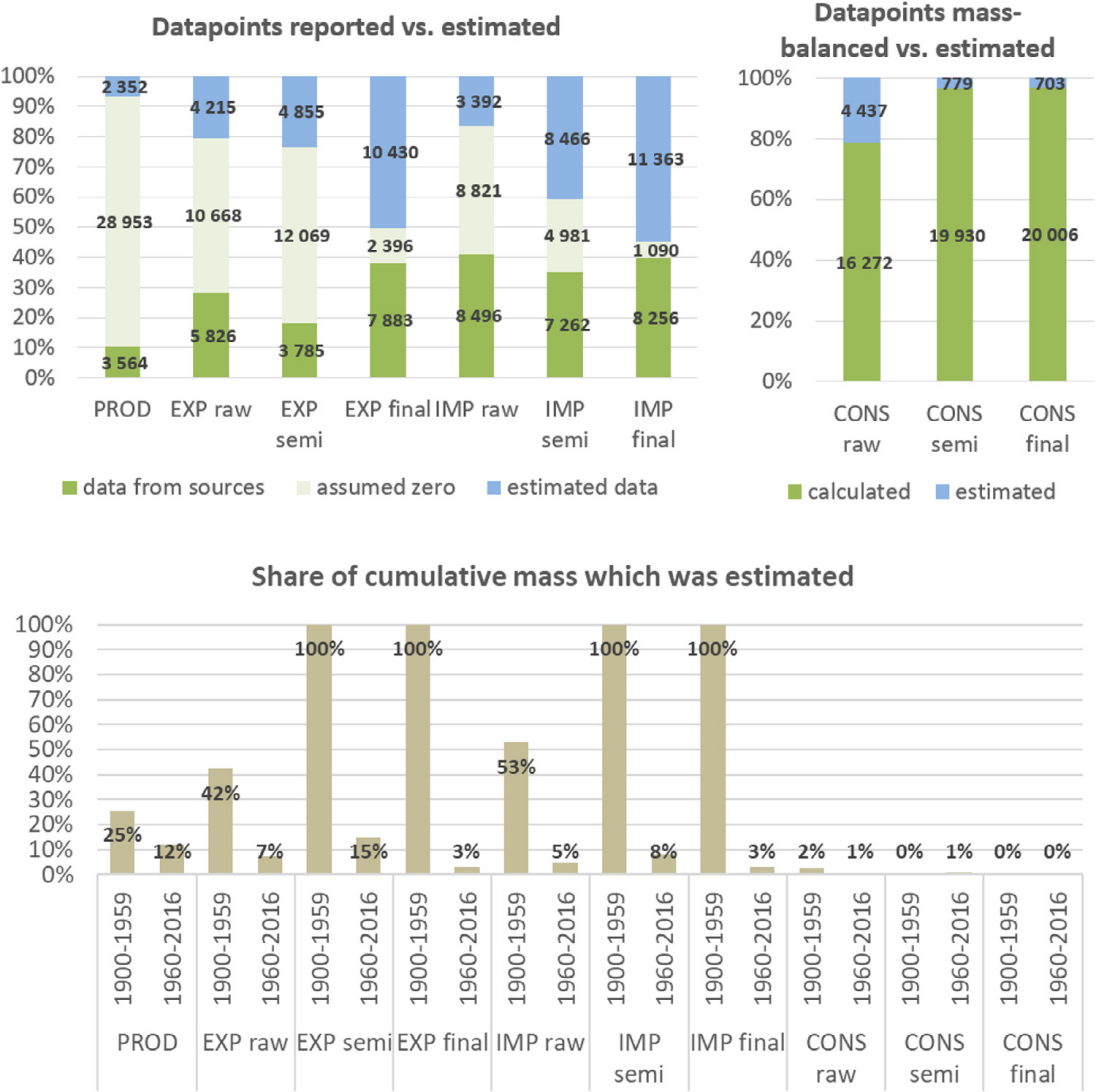
Counts on data from sources vs. estimated data for **lead**.

**Fig. 17:**
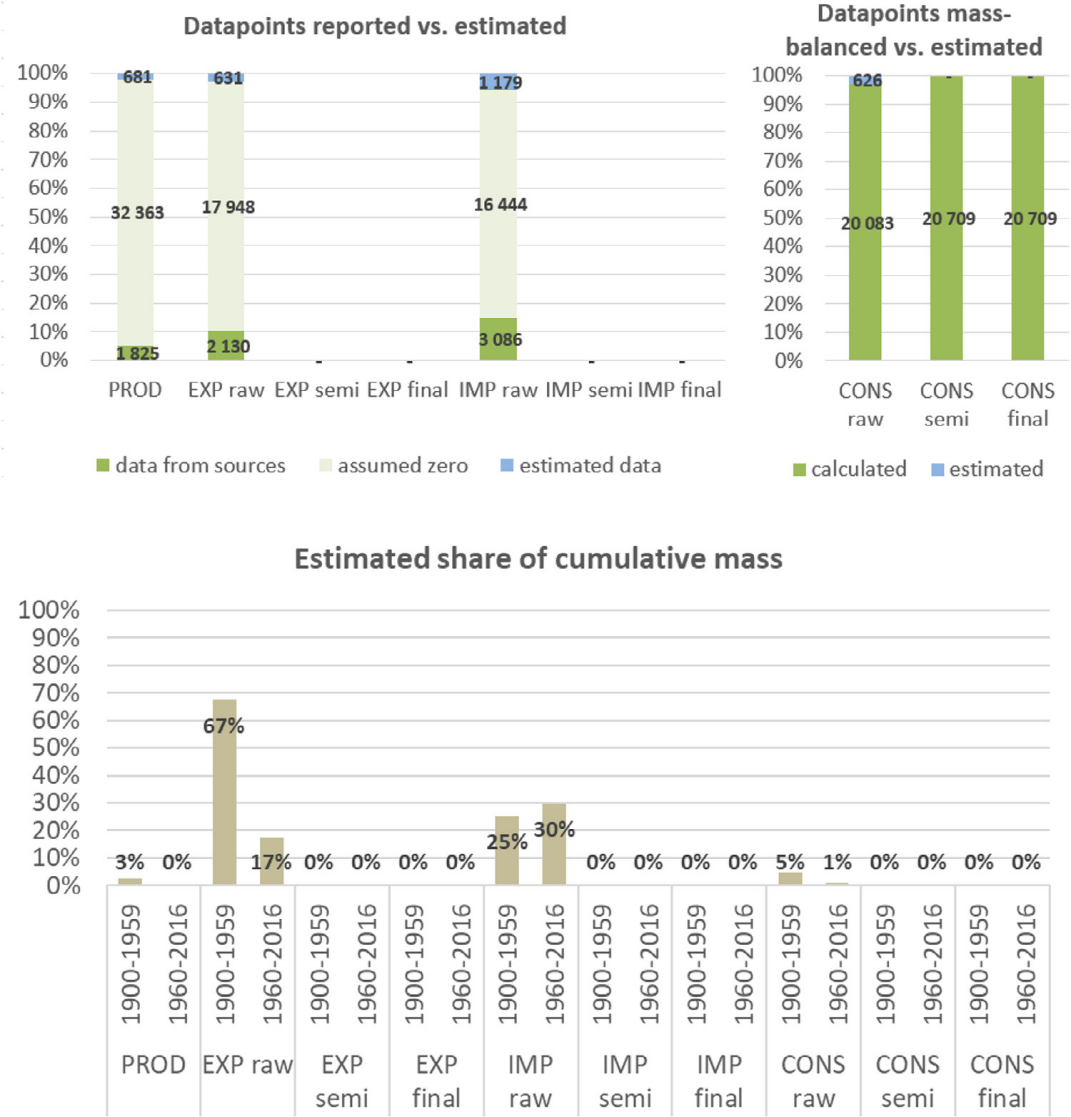
Counts on data from sources vs. estimated data for **other metals**.

**Fig. 18:**
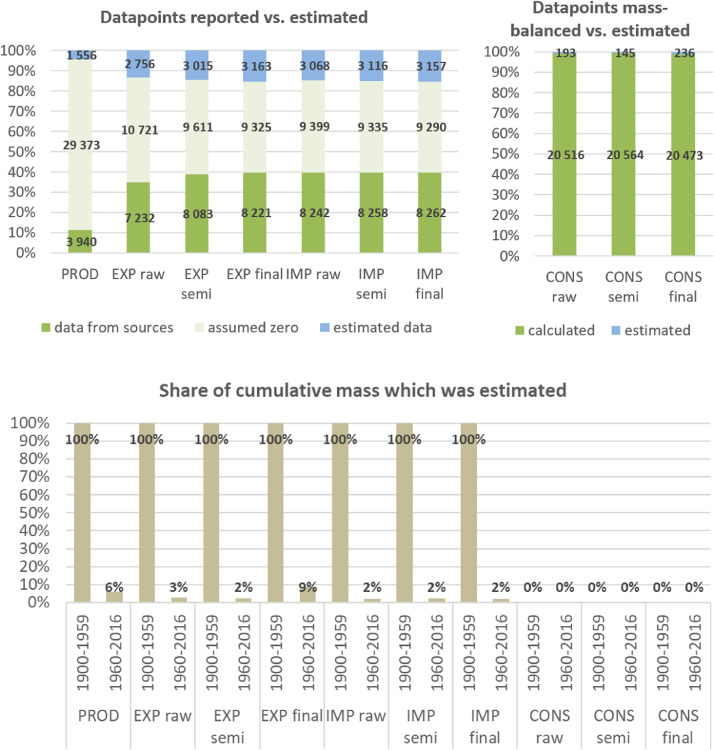
Counts on data from sources vs. estimated data for **plastics**.

**Fig. 19:**
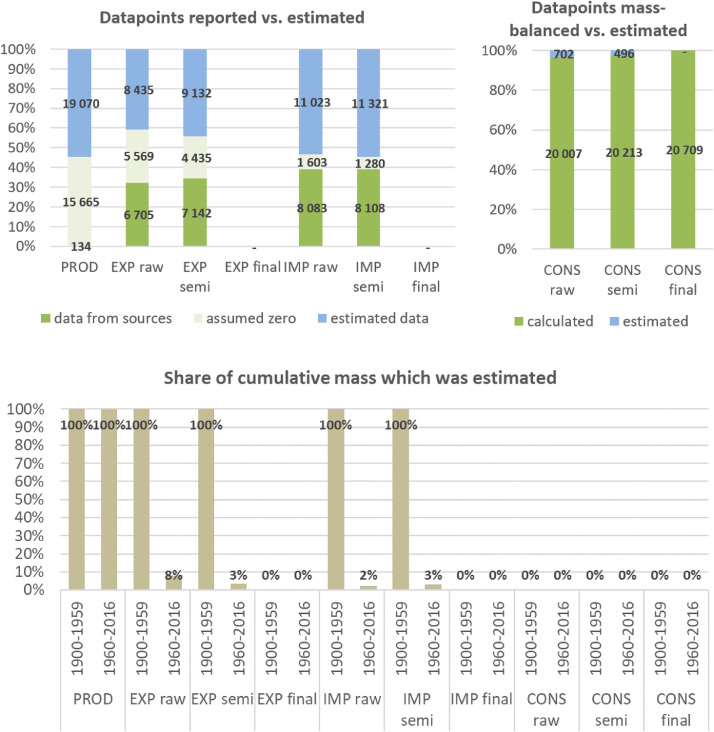
Counts on data from sources vs. estimated data for **container glass**.

**Fig. 20:**
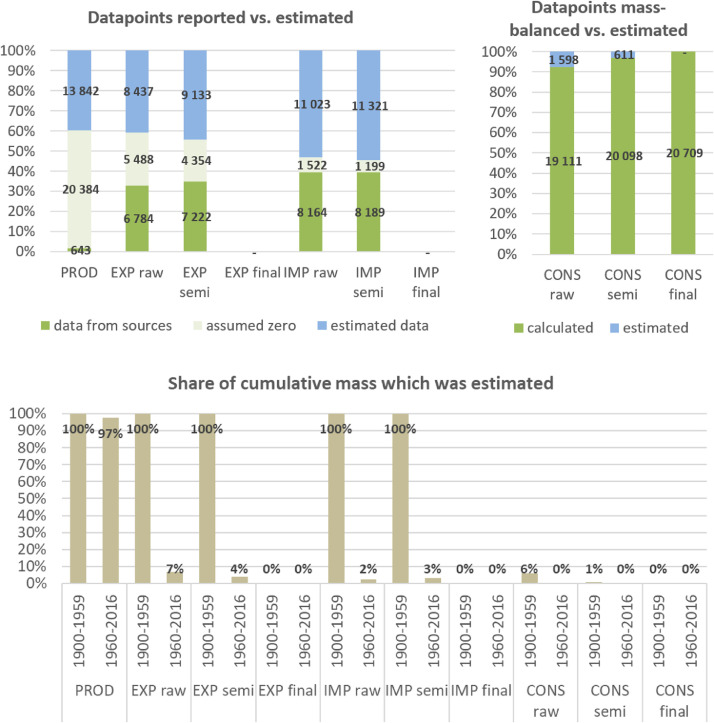
Counts on data from sources vs. estimated data for **flat glass**.

For almost all materials, GAS_prim_ results from Krausmann et al. [74] tend to be higher than the herein presented estimates (Fig. 5), whereas they align rather well for the extraction estimates (Fig. 6). For this novel database, huge efforts were undertaken to properly exclude double-counting, secondary material flows and processing wastes to arrive at a fully mass-balanced national-level dataset consistent with ew-MFA principles. These efforts are accountable for the differences to previous estimates, which were not compiled on the national level and are gathered in a less comprehensive way. Accounts for other metals differ especially strong, as only chromium, manganese, tin and nickel not used in steel alloys were included in the novel database to avoid double counting with steel accounts (see Section 6.6). For primary extraction of crude oil and natural gas, which is necessary for bitumen and plastics production, the novel estimates are largely higher than previous estimates from Krausmann et al., but align rather well with other estimates from the literature (e.g [47]). This is the case, as plastics production in Krausmann et al. [74] estimates were sourced from UNCPS data only, which is largely incomplete due to various reasons, wherefore we compiled data largely from the IEA database (see Section 6.7).

### 10.5 Results of the evaluation of data processing methods

In addition, it was surveyed how many of the datapoints have been estimated in comparison to the datapoints coming from data sources. We here distinguish between reported datapoints (incl. unit transformations), mass-balanced datapoints (calculated by adding or subtracting production and trade flows), plausibly assumed ‘zeros’ (when material flows are likely negligible) and estimated datapoints (all other estimation methods applied). We also calculated for each dataset how much of the total mass (tons) has been estimated as outlined above for each year. Estimation procedures considered here were interpolation, extrapolation, back-casting, country dissolution and outlier removals. Results for each material category are given in the graphs below.

### 10.6 Accumulated error from mass-balance corrections

**Fig. 21:**
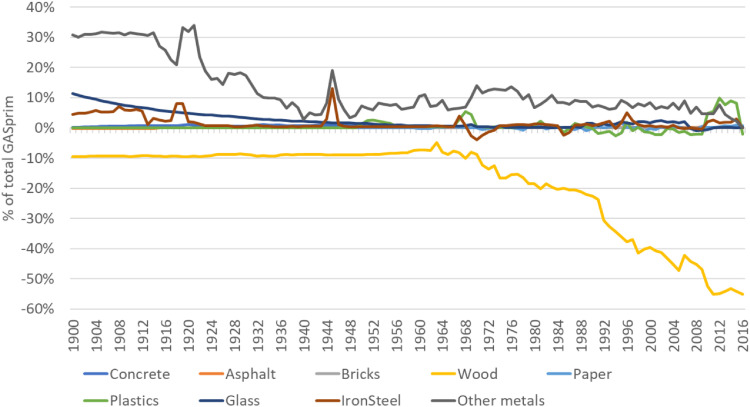
Total global accumulated error per material from all mass-balance corrections conducted as explained in Section 3/ Step 8.

**Fig. 22:**
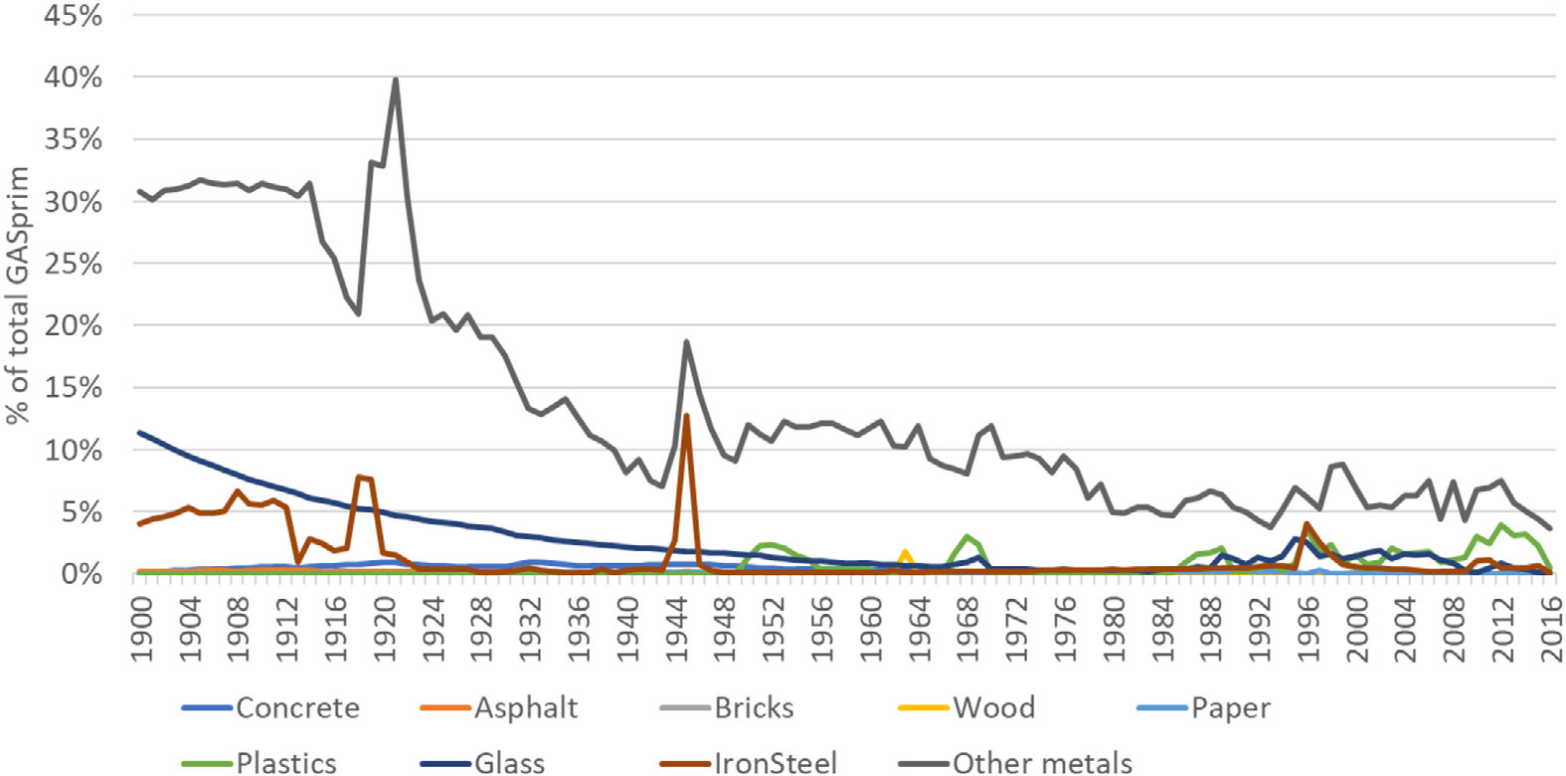
Global accumulated error per material for corrections of negative mass-balances conducted as explained in Section 3/ Step 8.

As explained in Section 3/ step 8, we corrected mis-matching mass-balances from production and trade data and the balancing of timber raw and semi-finished products and collected the corrected amounts in a global accumulated error account, which is shown in Fig. 21 and Fig. 22.

## CRediT authorship contribution statement

**Barbara Plank:** Conceptualization, Methodology, Validation, Formal analysis, Investigation, Data curation, Visualization, Writing – review & editing. **Jan Streeck:** Methodology, Visualization, Investigation, Writing – review & editing. **Doris Virág:** Investigation, Visualization, Writing – original draft. **Fridolin Krausmann:** Conceptualization, Methodology, Supervision, Writing – review & editing. **Helmut Haberl:** Conceptualization, Supervision, Funding acquisition, Writing – review & editing. **Dominik Wiedenhofer:** Conceptualization, Methodology, Visualization, Supervision, Writing – review & editing, Project administration.

## Declaration of Competing Interest

The authors declare that they have no known competing financial interests or personal relationships that could have appeared to influence the work reported in this paper.

## Appendix Supplementary materials

Image, application 1
